# Attention-deficit/hyperactivity disorder as a risk factor for being involved in intimate partner violence and sexual violence: a systematic review and meta-analysis

**DOI:** 10.1017/S0033291723001976

**Published:** 2023-12

**Authors:** Gonzalo Arrondo, Alfonso Osorio, Sara Magallón, Cristina Lopez-del Burgo, Samuele Cortese

**Affiliations:** 1 Institute for Culture and Society, University of Navarra, Pamplona, Spain; 2 School of Education and Psychology, University of Navarra, Pamplona, Spain; 3 School of Medicine, University of Navarra, Pamplona, Spain; 4 Centre for Innovation in Mental Health, School of Psychology, Faculty of Environmental and Life Sciences, University of Southampton, Southampton, UK; 5 Clinical and Experimental Sciences (CNS and Psychiatry), Faculty of Medicine, University of Southampton, Southampton, UK; 6 Solent NHS Trust, Southampton, UK; 7 Hassenfeld Children's Hospital at NYU Langone, New York University Child Study Center, New York City, New York, USA; 8 Division of Psychiatry and Applied Psychology, School of Medicine, University of Nottingham, Nottingham, UK

**Keywords:** ADHD, IPV, relationships, sexual violence, violence

## Abstract

**Background:**

Intimate partner violence (IPV) and sexual violence (SV) are significant problems world-wide, and they affect women disproportionally. Whether individuals with attention-deficit/hyperactivity disorder (ADHD) are at an increased risk of being involved in these types of violence is unclear.

**Methods:**

We carried out a systematic review and meta-analysis (PROSPERO registration CRD42022348165) of the associations between ADHD and being the victim or perpetrator of IPV and SV. Ratios of occurrence of violence were pooled in random-effects models and study risk of bias was evaluated using the Newcastle–Ottawa Scale.

**Results:**

A search on multiple databases, carried out on 7 October 2022, yielded 14 eligible studies (1 111 557 individuals). Analyses showed a higher risk of ADHD individuals being involved in IPV as perpetrators (six studies, OR 2.5, 95% CI 1.51–4.15) or victims (four studies, OR 1.78, 95% CI 1.06–3.0). Likewise, individuals with ADHD were at increased risk of being perpetrators (three studies, OR 2.73, 95% CI 1.35–5.51) or victims of SV (six studies, OR 1.84, 95% CI 1.51–2.24). Results were overall robust to different analytical choices.

**Conclusions:**

Individuals with ADHD are at an increased risk of being involved in cases of violence, namely IPV and SV, either as victims or perpetrators. Although the causal path or mediating variables for these results are still unclear, this increased risk should inform evidence-based psychoeducation with individuals with ADHD, their families, and partners about romantic relationships and sexuality.

## Introduction

Intimate partner violence (IPV) is defined as any kind of violence that takes place within a romantic relationship (World Health Organization, 2012). It includes physical, sexual, or psychological violence (Lopez-del Burgo, Osorio, de la Rosa, Calatrava, & de Irala, 2021). IPV is a significant problem around the world. Data from the Center of Disease Control and Prevention indicate that over 61 million women and 53 million men have experienced psychological aggression by an intimate partner in their lifetime in the USA (Leemis et al., 2022). IPV affects women disproportionally, not only in the USA, but also globally. According to the World Health Organization, 27% of women, aged 15–49 years, have experienced any type of IPV in their lifetime (Sardinha, Maheu-Giroux, Stöckl, Meyer, & García-Moreno, 2022). However, it must be noted that IPV is often bidirectional within the couple. Actually, the most frequent pattern of IPV in a couple is mutual violence (Straus, 2008, 2011). At the individual level, Lopez-del Burgo et al. (2021) found moderately positive correlations between suffered and perpetrated violence. Notably, the symmetry in perpetration is compatible with an asymmetry in the effects: women suffer more severe consequences of IPV (Straus, 2011).

Sexual violence (SV) has been defined as ‘a sexual act that is committed or attempted by another person without consent of the victim or against someone who is unable to consent or refuse’ (Breiding, Basile, Smith, Black, & Mahendra, 2015). SV may occur within a romantic relationship (and then it is a form of IPV) or outside it (when perpetrated by someone who has never been an intimate partner). SV is a widespread problem both within and outside romantic relationships. Non-partner SV has been reported by 0.3–12% of women above the age of 15 (García-Moreno, Jansen, Ellsberg, Heise, & Watts, 2005). Similarly to what it occurs for IPV, women are much more frequently sexually assaulted or abused than men. However, the rate of occurrence in men is also worrying. Data from the Global Burden of Disease study indicate that 4.2% of the female population and 2.4% of the male population has experienced at least one event of SV in the previous year (Institute for Health Metrics and Evaluation, 2019). Of note, IPV and SV are highly correlated. For example, half of the cases of SV occur within the couple (Black et al., 2011). Moreover, IPV and SV may share common risk factors.

Attention-deficit/hyperactivity disorder (ADHD) is a frequent neurodevelopmental disorder that affects around 3% of adults (Song et al., 2021). It is characterized by impairing, pervasive inattention and/or impulsivity and hyperactivity that entail a huge burden for the individual and society, including school and work under-achievement, worse physical health (Arrondo et al., 2022; Ruiz-Goikoetxea et al., 2018), and impaired social relations (Harpin, Mazzone, Raynaud, Kahle, & Hodgkins, 2016). Individuals with ADHD struggle with managing conflict and personal relationships and tend to have more tumultuous intimate relationships, with greater number of partners (Margherio et al., 2021), and higher rates of divorce (Klein et al., 2012), compared to neurotypical individuals.

Childhood ADHD is also a risk factor for antisocial behavior later in life, especially when comorbid with conduct problems (Mohr-Jensen & Steinhausen, 2016). Importantly, ADHD also increases the risk of being victimized by peers during the school years, with individuals with ADHD acting as perpetrators or being both victims and perpetrators (Fogler, Weaver, Katusic, Voigt, & Barbaresi, 2022). Currently, it is unclear if the pattern of having a higher risk of perpetrating or suffering violence extends to the domain of IPV and/or SV. Indeed, individuals with disabilities are frequent victims of IPV and SV. A recent meta-analysis examined the prevalence of IPV in women with disabilities. Significant associations were observed between disability and various forms of violence, including financial, physical, psychological, sexual, and any type of violence (García-Cuéllar, Pastor-Moreno, Ruiz-Pérez, & Henares-Montiel, 2023). Conversely, there is also meta-analytical evidence indicating that adults with disabilities, particularly those with mental illnesses, face an increased susceptibility to different types of violence, including SV, compared to their non-disabled counterparts (Hughes et al., 2012). There is some evidence that this also occurs in individuals with ADHD (Wymbs, Dawson, Egan, & Sacchetti, 2019), but this has not been systematically evaluated. It is also uncertain whether ADHD is related to higher rates of IPV or SV perpetration, which may be accounted for mainly by the impulsive component of the disorder. A systematic review carried out in January 2015 (Buitelaar, Posthumus, & Buitelaar, 2020) found some evidence that ADHD is a risk factor for IPV perpetration. However, it was beyond the scope of that review to include a statistical synthesis of the included studies, and several studies have been published since then. Evidence of a significant association of ADHD to IPV or to SV would be relevant to gain insights into these types of violence, and to inform preventive strategies them.

To fill this important gap, we systematically reviewed and meta-analyzed studies that assessed the association of ADHD to IPV or SV in adults or adolescents, either as victims or perpetrators of the violent acts.

## Methods

This systematic review/meta-analysis was registered in Prospero (CRD42022348165) and follows the 2020 PRISMA guidelines for systematic reviews (Page et al., 2021).

### Search

Searches were carried out in PubMed, Web of Science (Core Collection, Medline, Scielo) and Unika, an institutional database aggregator from the University of Navarra, Spain, that combines references over a hundred databases, based in the EBSCOhost service (detail on databases searched for this systematic review can be found in online Supplementary Table S1). The search syntax, which was adapted for the different databases, combined terms on ADHD, IPV and SV (Full search keys are included in online Supplementary Table S2). The final search was carried out in 7 October 2022, with no date or language restrictions. References of relevant systematic reviews were hand searched to retrieve any additional eligible study. Inverse search in Google Scholar of articles citing them was also used to find additional potentially eligible articles, as was the screening of publications lists of key authors in the field (authors in two or more of the included studies).

### Inclusion criteria

We included observational studies (cross-sectional, prospective, or retrospective) assessing the relationship between ADHD and the risk of IPV or SV, as either perpetrator or victim. Violence and ADHD had to be defined dichotomously. The following ways to define ADHD were accepted: (1) a clinical diagnosis, based on standardized criteria, (2) a score over a threshold in a validated symptom scale, (3) a report of ADHD diagnosis or stimulant prescription in medical or administrative files/registries and (4) a positive answer to the question: ‘Did your doctor ever tell you that you have ADHD?’ or similar. The latter was included as it permits the inclusion of large-scale surveys where self-reported diagnoses are typically the only indication of a given mental or physical disorder, and it is a frequently accepted method for the identification of cases in meta-analyses. Studies were retained regardless of the medication status of the included participants with ADHD and the setting where they were recruited (i.e. general population or clinic).

Regarding IPV and SV, we accepted any definition provided by study authors. We included studies of IPV in general or on specific subtypes (e.g. physical, psychological or sexual). We retained studies on SV regardless of the type of relationship (romantic or not) between victim and aggressor. It is common in the literature to include psychological, physical, and SV as a unitary construct, as most predictors of IPV are associated with all types of IPV (Lopez-del Burgo et al., 2021). For example, Scherer, Snyder, and Fisher (2016) studied the three types as a whole and also provided a review on many previous studies that used a similar approach. Additionally, the different types of violence should be assessed separately if possible. Due to the expected small number of studies a joint analysis for all types of IPV, and another analysis for SV (in both cases separating perpetration and victimization) were carried out. Regarding age, violence had to occur at any point during adolescence (over 13 years old) or adulthood (above 18 years old). Hence, violence in childhood (less than 13 year-old) was excluded. Studies including individuals of any sex were eligible.

We evaluated the association between ADHD and four key outcomes: IPV as victim, SV as victim, IPV as perpetrator and SV as perpetrator. These associations had to be expressed as odds ratio (OR), risk ratio (RR), hazard ratio (HR) or equivalent. We prioritized adjusted ORs over other types of effect sizes. If unavailable, we also used (in order of priority) (1) adjusted HRs, (2) unadjusted ORs, (3) unadjusted HRs or RRs. We used the most adjusted (i.e. maximally adjusted) ratios in the analyses among those reported in the included studies.

### Screening, data extraction and risk of bias assessment

References from each database were exported to Mendeley, where duplicates were first removed automatically. After that, any residual duplicate was removed manually. Then, titles and abstracts were screened to determine the suitability of each document. Full texts of documents of interest were retrieved to judge the final inclusion. The following data were extracted from eligible articles: (1) Publication details: year and language of publication, country where the study was conducted; (2) Design: type of study (e.g. cross-sectional, case–control, cohort); (3) study temporality (cross-sectional, prospective, retrospective); (4) patient enrollment (consecutive, non-consecutive); (5) setting (clinical *v.* population-based study); (6) study participants details: number, mean age (s.d.), sex distribution, socioeconomic status and ethnicity of participants with and without ADHD or history of violence; (7) psychiatric comorbidities; (8) method to establish the identification of ADHD and violence; (9) measure of association (OR, RR, HR or equivalent); (10) rate of events within the exposed and unexposed participants-this was used to obtain the unadjusted OR whenever it was not provided.

Specifically, we extracted the following effect sizes from the studies (if available), both as a perpetrator and as a victim: association between ADHD and (1) SV in general, (2) IPV in general and (3) subtypes of IPV (psychological, physical and sexual).

Risk of bias of the studies was assessed with the Newcastle–Ottawa Scale (NOS) (Wells et al., 2000), which was adapted to the specificities of the current project (online Supplementary Tables S3–S5). This scale evaluates observational studies on three different domains, namely: (1) selection, (2) comparability, and (3) exposure. It has a maximum score of nine stars, and any study with a score of six or less was considered to be at a high risk of bias (Xun et al., 2023). The NOS has been modified in the literature to accommodate case–control, cohort and cross-sectional studies (Xun et al., 2023).

All steps were carried out independently by two authors, and consensus was reached whenever there was a disagreement.

### Data synthesis

ORs or other ratios expressing the association between violence and ADHD were extracted or calculated from available data. We conducted four main analyses on the maximally adjusted ratios, in relation to: the association between ADHD and suffering IPV, the association between ADHD and suffering SV, the association between ADHD and perpetrating IPV and, lastly, the association between ADHD and perpetrating SV.

Meta-analyses used random-effects models and were weighted by the reciprocal of the variance of the effect size with a Restricted Maximum-Likelihood estimator. We used *Q*, Tau squared (*p* < 0.1 was considered significant due the low power of the test) (Higgins, Thompson, Deeks, & Altman, 2003), and the *I*^2^ index to assess the heterogeneity of effect sizes. We used Egger's and Begg's tests to assess publication/small-sample bias (*p* < 0.1 was considered significant due the low power of the tests (Simmonds, 2015)). Analyses were performed using Stata v17.

Per protocol, we planned to assess the feasibility of conducting several subgroup meta-analyses (online Supplementary Table S6). We operationalized this by only running analyses for which at least three studies were available. Due to data availability, we were finally able to carry out the following analyses: studies on females (for SV as victim), physical IPV (as victims or perpetrators), cross-sectional studies only (for IPV as perpetrator and SV as victims), including population-based studies only (for IPV as perpetrator and SV as victims), including only studies where ADHD diagnosis was confirmed clinically or through an administrative register (for IPV as perpetrator), and including only adjusted OR (for the four key analyses).

We also run a post-hoc leave-one-out analysis to assess the impact of specific papers in our estimations. As the number of studies was low, the specificities of individual studies could have a relevant effect. Similarly, we excluded studies that were carried out on samples within the legal system (arrests, accused or convicts) in an additional post-hoc sensitivity analysis.

## Results

**Figure 1.**
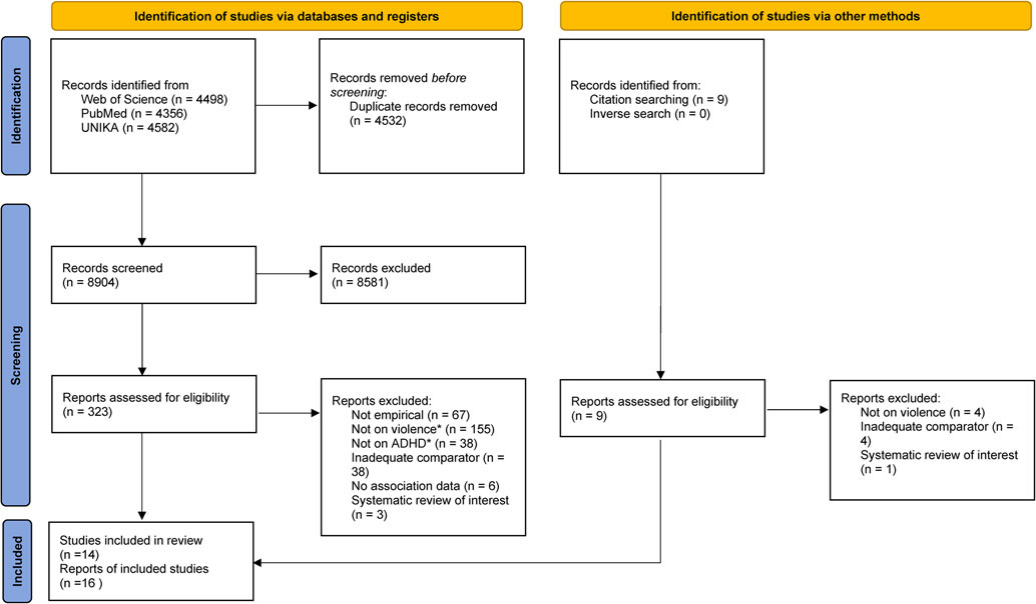
PRISMA chart.

Out of an initial pool of 8904 potentially eligible records, 14 unique studies fulfilled our inclusion criteria (Blocher et al., 2001; Campe, 2021; Crane, Hawes, Devine, & Easton, 2014; Fang, Massetti, Ouyang, Grosse, & Mercy, 2010; González, Kallis, & Coid, 2013; Guendelman, Ahmad, Meza, Owens, & Hinshaw, 2016; McCauley, Breslau, Saito, & Miller, 2015; Mohr-Jensen, Müller Bisgaard, Boldsen, & Steinhausen, 2019; Ngo, Veliz, Kusunoki, Stein, & Boyd, 2018; Scherer et al., 2016; Snyder, 2015; Wymbs & Gidycz, 2021; Wymbs et al., 2012; Yu et al., 2019). Figure 1 shows the screening and selection of studies. Online Supplementary Table S7 reports the reason for exclusion of any studies discarded after checking its full text. Online Supplementary Table S8 details the included studies reported in multiple records (papers).

The characteristics of the included studies can be found in Table 1 and online Supplementary Tables S9–S13. All studies but one had been published in English, all were published between 2001 and 2021, and included individuals from the United States (*k* = 8) or Europe (*k* = 4). The combined sample across studies included 1 111 557 individuals, with a median sample size of 14 816 (range: 218–1 025 450). Most studies (*k* = 9) were cross-sectional, two were cohort studies, and one was a case–control study. The majority of the studies included adolescents or young adults. ADHD was most frequently identified with symptom scales (*k* = 5), but also through self-reports (*k* = 3), clinical diagnoses (*k* = 2) or information in registers (*k* = 2). Violence was typically assessed through self-reported questionnaires, but legal registries were also used (*k* = 3). Risk of bias was apparent in most studies, with nine studies having six or less stars in our evaluation with the NOS (online Supplementary Table S14). Ascertainment of exposure (ADHD) was the most critical item, as 65% of the studies were considered to be at high risk of bias.

**Table 1.** Characteristics of studies included in the systematic review

Reference	Country	Database	Design	Setting	Diagnosis of ADHD	Identification of violence	*N*	Age	Sex (% male)
Blocher et al. (2001)	Germany	NA	C-C	Other	Scale	Convictions	294	NR	100
Campe (2021)	US	ACHA-NCHA-2016	C-S	Population based	Self-report	Self-report	22 828	NR	0
Crane et al. (2014)	US	NA	C-S	Other	Clinical	Self-report	190	*M* = 30.44 (s.d. = 10); 18-59	66
Fang et al. (2010)	US	Add Health	C-S	Population based	Scale	Self-report	11 238	*M* = 22	49.6
González et al. (2013)	England	APMS, 2007	C-S	Population based	Scale	Self-report	7369	NR	NR
Guendelman et al. (2016)	US	BGALS	Cohort	Other	Clinical	Self-report	193	*M* = 9.5 (s.d. = 1.7) at diagnosis, with 10 year follow up	0
McCauley et al. (2015)	US	NCS-R	C-S	Population based	Clinical	Self-report	5112	>21 (dating before age 21)	NR
Mohr-Jensen et al. (2019)	Denmark	DPCRR and DNPR	Cohort	Population based	Register	Convictions	23 826	NR	NR
Ngo et al. (2018)	US	SSLS	C-S	Population based	Scale	Self-report	2282	12–18	100
Scherer et al. (2016)	US	ACHA-NCHA II-2008	C-S	Population based	Self-report	Self-report	20 486	*M* = 19.63 (s.d. = 1.56); 18-25	30.89
Snyder (2015)	US	ACHA-NCHA II-2008	C-S	Population based	Self-report	Self-report	14 816	*M* = 19.6; 18–24	0
Wymbs et al. (2012)	US	PALS; STP	C-S	Other	Clinical	Self-report	231	18–25	100
Wymbs et al. (2021)	US	MTurk	C-S	Population based	Scale	Self-report:	218	18–45	55%
Yu et al. (2019)	Sweden	Swedish national registries	Cohort	Population based	Register	Arrests	1 025 450	NR	NR

NA, Not applicable; NR, not reported; C-C, case–controlled; C-S, cross-sectional. See online Supplementary material for additional data.

Seven studies were on IPV, five studies on SV, and two studies on IPV, but reported SV within the couple as an outcome and, hence, could be included in both analyses. Effect size data can be found in online Supplementary Tables S15 and S16.

**Figure 2.**
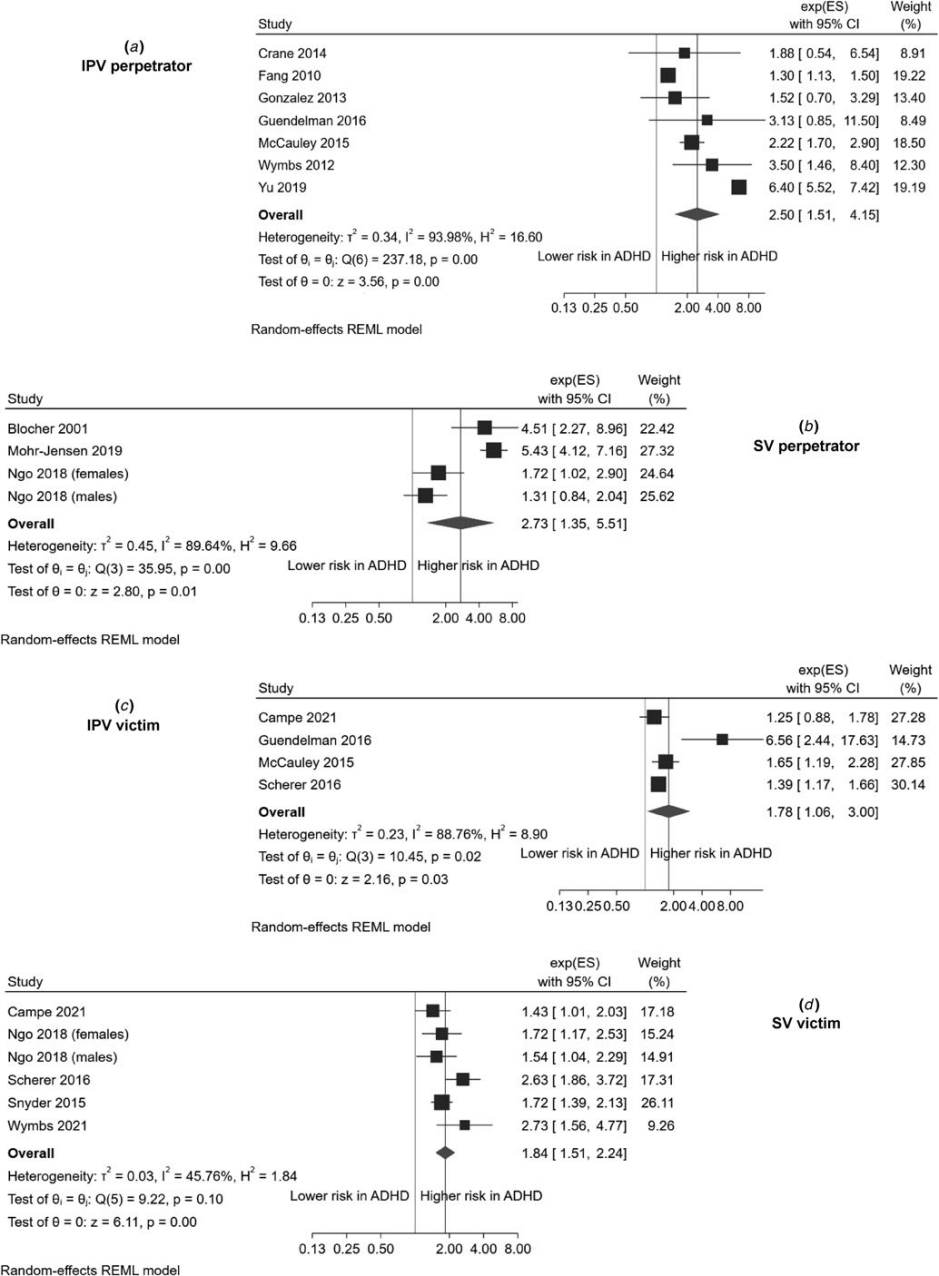
Forest plots showing the results of the main meta-analyses.

The analysis with the largest number of studies was on ADHD individuals being involved in IPV as perpetrators (six studies, pooled sample: 1 049 598 individuals). Results for this analysis are shown in Fig. 2*a*, Table 2 and online Supplementary Fig. 1. The summary effect indicated that individuals with ADHD were significantly more likely to commit this type of violence (OR 2.5, 95% CI 1.51–4.15), albeit the analysis was characterized by significant heterogeneity (*Q* = 237.18, *p* < 0.001; *I*^2^ = 93.98%). The effect stayed significant through the leave-one-out and all the other sensitivity and subgroup analyses (Table 2, online Supplementary Figs S2–S14). However, the overall effect size was greatly reduced (OR 1.87, 95% CI 1.32–2.66) with the elimination of the biggest study.

**Table 2.** Summary of main, sensitivity and subgroup analyses

Analysis	*K*	*N*	Effect size (95% CI)	Heterogeneity				Egger's test			Begg's test		
				*Q*	*p*	*I*^2^	*T*^2^	*B*	SE	*p*	*K*	SE	*p*
Main analyses													
IPV perpetrator	7	1 049 598	2.5 (1.51–4.15)	237.18	<0.001	93.98	0.34	−0.2	1.22	0.873	7	6.66	0.368
SV perpetrator	4	28 785	2.73 (1.35–5.51)	35.95	<0.001	89.64	0.45	−1.01	5.72	0.859	0	2.94	1
IPV victim	4	48 619	1.78 (1.06–3)	10.45	0.015	88.76	0.23	3.38	1.33	0.011	2	2.94	0.734
SV victim	6	62 871	1.84 (1.51–2.24)	9.22	0.100	45.76	0.03	1.82	2.21	0.411	1	5.32	1
Sugroup and sensitivity analyses													
IPV physical perpetrator	5	23 935	1.72 (1.22–2.43)	13.49	0.009	64.88	0.07	0.64	0.91	0.48	4	4.08	0.462
IPV perpetrator; cross sectional	5	23 996	1.81 (1.26–2.6)	15.92	0.003	70.4	0.09	0.83	1.02	0.415	2	4.08	0.806
IPV perpetrator; population-based	4	46 547	1.58 (1.19–2.11)	12.06	0.007	71.96	0.05	0.22	1.7	0.899	2	2.94	0.734
IPV perpetrator; clinical or register-based diagnosis	4	1 030 839	3.39 (1.84–6.26)	49.05	<0.001	90.01	0.29	−1.21	1.56	0.436	−2	2.94	0.734
IPV perpetrator; no convicts	5	24 084	1.88 (1.32–2.66)	17.81	0.001	72.58	0.09	1.09	1.07	0.307	0	4.08	1
IPV perpetrator; adjusted	5	1 048 703	2.35 (1.2–4.63)	186.58	<0.001	96	0.5	−0.25	1.87	0.895	2	4.08	0.806
IPV physical victim	3	25 791	2.7 (1.39–5.22)	9.51	0.009	86.7	0.27	3.01	1.94	0.121	1	1.91	1
IPV victim; adjusted	3	48 426	1.41 (1.23–1.63)	1.36	0.505	0	0	0.21	2.32	0.928	−1	1.91	1
SV victim; females	3	39 885	1.65 (1.4–1.95)	0.84	0.659	0	0	−1.03	2.17	0.634	−1	1.91	1
SV victim; cross sectional	4	40 002	1.69 (1.44–1.98)	3.95	0.267	0	0	1.77	2.06	0.391	2	2.94	0.734
SV victim; population-based	4	40 002	1.69 (1.44–1.98)	3.95	0.267	0	0	1.77	2.06	0.391	2	2.94	0.734
SV victim; adjusted	3	27 493	1.55 (1.25–1.92)	0.48	0.786	0	0	5.42	10.5	0.606	1	1.91	1

We found only three studies (combined sample 28 785 individuals) assessing the association between ADHD and perpetrating SV, and the pooled OR across these studies indicated a significant association (OR 2.73, 95% CI 1.35–5.51) but with important heterogeneity (*Q* = 35.95, *p* < 0.001; *I*^2^ = 89.64%). Results for this analysis are shown in Fig. 2*b*, Table 2 and online Supplementary Fig. S15. The overall effect was highly sensitive to the elimination of specific studies (online Supplementary Fig. S16). However, due to the low number of studies, no additional sensitivity or subgroup analyses could be carried out.

Our results also indicated that individuals with ADHD were more frequently victims of IPV than individuals without ADHD (four studies, pooled sample 48 619 individuals; OR 1.78, 95% CI 1.06–3.0), with significant heterogeneity among the studies (*Q* = 10.45, *p* = 0.015; *I*^2^ = 88.76%). Results for this analysis are shown in Fig. 2*c*, Table 2, and online Supplementary Fig. S17. The leave-one-out analysis had mixed results (online Supplementary Fig. S18): pooled effect sizes did not shift greatly, but confidence intervals in three cases were not statistically significant anymore. The other sensitivity or subgroup analyses that could be carried out did not materially change the overall result (Table 2, online Supplementary Figs S19–S22).

Finally, ADHD individuals were also at a higher risk of suffering SV (six studies, pooled sample 62 871 individuals). Results for this analysis are shown in Fig. 2*d*, Table 2 and online Supplementary Fig. S23. The summary effect yielded an OR of 1.84, (95% CI 1.51–2.24), with some heterogeneity (*Q* = 9.22, *p* = 0.1; *I*^2^ = 45.76%). The leave-one-out analysis showed that the effect was very robust to the elimination of specific studies (online Supplementary Fig. S24). Likewise, the different sub-analyses that could be carried out did not affect the overall results (Table 2, online Supplementary Figs S25–S32).

## Discussion

To our knowledge, this is the first systematic review and meta-analysis on the link between ADHD and either SV or IPV in adolescents and adults. Our analyses showed a significantly increased risk for individuals with ADHD of being involved in cases of violence. The association was statistically significant for the four outcomes analyzed: individuals with ADHD had a greater risk of perpetrating SV or IPV and, importantly, were at a greater risk of suffering SV or IPV.

Our results on IPV are consistent with evidence that individuals with ADHD tend to have poorer intimate relationships and higher rates of ruptures (Klein et al., 2012; Margherio et al., 2021; Wymbs, Canu, Sacchetti, & Ranson, 2021). Adults with ADHD tend to be less satisfied in their relationships than individuals without ADHD. Conversely, the romantic quality of their partners is also lower, with almost all individuals indicating that symptoms interfere in the relationship (Eakin et al., 2004; Wymbs et al., 2021). Moreover, ADHD is characterized as a disorder of self-regulation that includes control difficulties over the emotional domain (de la Fuente et al., 2019). This combination of frustration building and poor behavioral and emotional control could contribute to the relation between ADHD and IPV, both as victim and perpetrator. On the one hand, the core symptoms of ADHD could put individuals at risk of being victimized by individuals without ADHD but with violent tendencies. On the other hand, hyperactivity, impulsivity and emotional dysregulation could facilitate the victimization of a partner by some individuals with ADHD. Of note, the roles of victim and perpetrator in a relationship are not incompatible. Specifically, a study in 32 countries found that around 70% of couples who reported violence, and around 58% of couples who reported severe violence, were couples with bidirectional violence (where both partners were violent) (Straus, 2008). However, as the included studies did not report how many participants were both victims and perpetrators, we could not specifically assess this aspect. Whether this overlap is greater in ADHD than in the general population remains an open question.

Our second result, namely that ADHD is also a risk factor both for being the victim or the perpetrator of acts of SV during adolescence or adulthood, is more challenging to explain. We can hypothesize on the possible mechanisms leading to this effect. First, this could be partly explained by the fact that SV occurs mainly within couples (Spencer et al., 2023). The fact that SV in two of the included studies occurred solely in the context of IPV provides support to this hypothesis. If ADHD increases the risk of being involved in IPV, it could secondarily also increase the risk of SV. Second, there could be mediating factors related to ADHD as well as to perpetrating or suffering SV. An example of these type of factors could be alcohol intake. In fact, individuals with ADHD tend to drink more (Elkins et al., 2018), and in turn, alcohol intake has also been linked to an increase risk of being a sexual victim or perpetrator (Spencer et al., 2023). Of note, this explanation could also be applied to the results on IPV, as it has been shown that alcohol or drugs intake moderates the risk of IPV perpetration or victimization (Wymbs et al., 2021). Third, there could be a differential influence of impulsivity/hyperactivity and inattentiveness by sex. Impulsivity and poor self-control have been related to violent acts (Yu et al., 2021), whereas inattentiveness might put individuals at a greater risk of being victimized in general (Aguado-Gracia et al., 2021). Since inattentive symptoms are more common in female individuals with ADHD, future studies might show an interaction between sex and role (victim/perpetrator) within violence.

When comparing the four types of outcomes, effect sizes were higher for ADHD as a risk factor for perpetration of violence than for victimization. However, these results were sensitive to the elimination of specific studies and the difference was not tested statistically. Moreover, in the case of the risk of SV perpetration, the number of studies was small and included studies that identified sexual offenders within the legal system, and hence might not generalize to the general population. Regarding IPV perpetration, discarding the studies derived from the legal system (which was also the biggest of the included studies) reduced the effect size, but the association remained statistically significant.

Our systematic review and meta-analysis should be considered in the light of some limitations, mainly due to the limitations of the included studies rather than of our work. The number of included studies was relatively small, albeit they generated a large sample size. While a meta-analysis with large sample sizes can provide a robust pooled effect size, the relatively limited number of studies in the current meta-analysis restricts the external validity and generalizability of the findings, as they might be driven by specific characteristics of the individual studies. Whereas heterogeneity was high in all our sets of results, very few of our planned sensitivity analyses could be carried out. Hence, heterogeneity could not be satisfactorily accounted for which complicates the interpretation of our summary statistics. Our results suggest a presence of diverse factors or sources of variation that influence the outcome of interest, but these factors could not be pin-pointed. Therefore, it is unclear to which degree the increased risks of IPV and SV apply to the whole sample of individuals of ADHD or derive from specific methodological choices of the studies on the issue. Among the variables that could not be adequately controlled, sex was especially relevant. Indeed, sex is a key variable explaining the frequency of ADHD in a sample. Moreover, violence perpetration *v.* victimization is also related to sex. Study designs made the evaluation of this relation even harder: studies on ADHD as a risk factor for being a victim of violence tended to be carried out solely in women, whereas studies on perpetrators tended to the opposite. However, it is important to note that this methodological characteristic controls for sex variables to some extent, as studies will compare individuals of the same sex with and without the disorder. Therefore, our results may provide stronger evidence for a greater risk of aggression on ADHD males and of victimization in ADHD females. Further studies are required to test whether these risks hold for the opposite sex.

Comorbidity of ADHD with mental disorders, including, among others, childhood conduct disorder, antisocial personality disorder, alcohol or drug abuse, could also be driving some of our results. Indeed, there is preliminary evidence for the role of antisocial personality and excessive alcohol intake as moderating the relationship between ADHD and IPV perpetration in males (Wymbs & Gidycz, 2021; Wymbs et al., 2017), and rates of comorbid mental disorders tended to be greater among individuals with ADHD. However, it is important to note that the aim of our study was descriptive. Regardless of possible causal effects, our results point towards some individuals with ADHD having an increased risk of violence, and we deem that this is of clinical relevance by itself.

The effect of ADHD medication could not be evaluated either, as this would have required access to individual patient data, which was beyond the scope of the present work. There is evidence that stimulants reduce criminality rates (Lichtenstein et al., 2012), and they could also have a similar effect on violence perpetration. They also reduce exposure to risky situations and accidents (Ruiz-Goikoetxea et al., 2018), and might prevent victimization to some extent. However, this limitation would bias results towards the null hypothesis. If participants in the included studies were all under ADHD medication, this would expectedly reduce the odds of violence within the ADHD group. We may then suspect that, without medication, effect sizes would be even larger than the ones we have found. Additionally, risk of bias was also present in most studies. Only three were judged to have a low risk of bias according to the NOS. Finally, statistical tests used for the assessment of publication bias are not highly sensitive when used with few (less than 10) studies. Hence, their negative results should be taken with caution.

These limitations of the literature call for future studies that can further our understanding of the relationship between ADHD, violence and its moderators. Indeed, future studies should adequately address differences between men and women and across the life-span. Similarly, the effect of moderating variables such as current or past comorbidity with conduct disorder or alcohol and substance abuse should be taken into account. Moreover, additional studies may provide further insight in specific variables driving the heterogeneity in meta-analyses.

Our results are of high clinical and social relevance but could be misinterpreted. On the one hand, we must emphasize that we have presented relative risks of an infrequent event. Therefore, our results should not be used to stigmatize individuals with ADHD, as most individuals with the disorder will not be involved in a case of IPV or sexual victimization. On the other hand, the fact that ADHD is related to a significantly higher risk of perpetrating or of being the victim of violence should not be minimized, as IPV and SV can have lasting consequences in the person who suffers them. Results should inform evidence-based psychoeducation with individuals with ADHD, their families and partners about relationships and sexuality. By acknowledging that ADHD can increase the risk of being involved in a case of IPV or SV, but also that this risk is moderated by many other contextual or personal factors, we can help individuals to be more aware of violence-leading situations and prevent them.

## Supporting information

Arrondo et al. supplementary material Arrondo et al. supplementary material

## Supplementary material

The supplementary material for this article can be found at https://doi.org/10.1017/S0033291723001976.
