# Supplementary material for: Attention-deficit/hyperactivity disorder as a risk factor for being involved in intimate partner violence and sexual violence: a systematic review and meta-analysis
Source: Psychol Med. 2023 Jul 24;53(16):7883–92. doi: 10.1017/S0033291723001976 (PMC10755239; doi:10.1017/S0033291723001976)

Attention-deficit/hyperactivity disorder as a risk factor for being involved in intimate partner violence and sexual violence: a systematic review and meta-analysis

Supplementary material

# Supplementary Tables index

[**Table S1 Providers in UNIKA (University of Navarra bibliographic aggregator) with at least 10 results** 3](#_Toc137455668)

[**Table S2 Search strategy** 5](#_Toc137455669)

[**Table S3 Operationalization of the Newcastle-Ottawa scale for case-control studies** 7](#_Toc137455670)

[**Table S4 Operationalization of the Newcastle-Ottawa scale for cohort studies** 8](#_Toc137455671)

[**Table S5 Operationalization of the Newcastle-Ottawa scale for cross-sectional studies** 9](#_Toc137455672)

[**Table S6 List of planned subgroup meta-analyses and whether they were finally carried out** 10](#_Toc137455673)

[**Table S7 References excluded after full-text evaluation with reasons for exclusion** 11](#_Toc137455674)

[**Table S8 Studies reported over multiple publications** 52](#_Toc137455675)

[**Table S9 Details on the databases used in the studies** 53](#_Toc137455676)

[**Table S10 Details on setting, identification of ADHD and identification of violence in the studies** 54](#_Toc137455677)

[**Table S11 Details on the overall samples of the studies** 57](#_Toc137455678)

[**Table S12 Data on the ADHD samples** 60](#_Toc137455679)

[**Table S13 Data on the non-ADHD sample (ADHD comparators)** 63](#_Toc137455680)

[**Table S14 Risk of bias evaluation** 66](#_Toc137455681)

[**Table S15 Effect size data** 67](#_Toc137455682)

[**Table S16 Effect sizes; analyses index** 70](#_Toc137455683)

# Supplementary Figures index

[**Figure S1 IPV perpetrator; funnel plot** 73](#_Toc137455684)

[**Figure S2 IPV perpetrator; leave-one-out analysis** 73](#_Toc137455685)

[**Figure S3 IPV physical perpetrator; forest plot** 74](#_Toc137455686)

[**Figure S4 IPV physical perpetrator; funnel plot** 74](#_Toc137455687)

[**Figure S5 IPV perpetrator; cross sectional; forest plot** 75](#_Toc137455688)

[**Figure S6 IPV perpetrator; cross sectional; funnel plot** 75](#_Toc137455689)

[**Figure S7 IPV perpetrator; population-based; forest plot** 76](#_Toc137455690)

[**Figure S8 IPV perpetrator; population-based; funnel plot** 76](#_Toc137455691)

[**Figure S9 IPV perpetrator; clinical or register-based diagnosis; forest plot** 77](#_Toc137455692)

[**Figure S10 IPV perpetrator; clinical or register-based diagnosis; funnel plot** 77](#_Toc137455693)

[**Figure S11 IPV perpetrator; adjusted; forest plot** 78](#_Toc137455694)

[**Figure S12 IPV perpetrator; adjusted; funnel plot** 78](#_Toc137455695)

[**Figure S13 IPV perpetrator; without studies deriving samples from the legal system; forest plot** 79](#_Toc137455696)

[**Figure S14 IPV perpetrator; without studies deriving samples from the legal system; funnel plot** 79](#_Toc137455697)

[**Figure S15 SV perpetrator; funnel plot** 80](#_Toc137455698)

[**Figure S16 SV perpetrator; leave-one-out analysis** 80](#_Toc137455699)

[**Figure S19 IPV victim funnel; funnel plot** 81](#_Toc137455700)

[**Figure S20 IPV victim; leave-one-out analysis** 81](#_Toc137455701)

[**Figure S21 IPV physical victim; forest plot** 82](#_Toc137455702)

[**Figure S22 IPV physical victim; funnel plot** 82](#_Toc137455703)

[**Figure S23 IPV victim; adjusted; forest plot** 83](#_Toc137455704)

[**Figure S24 IPV victim; adjusted; funnel plot** 83](#_Toc137455705)

[**Figure S25 SV victim funnel; funnel plot** 84](#_Toc137455706)

[**Figure S26 SV victim; leave-one-out analysis** 84](#_Toc137455707)

[**Figure S27 SV victim; females; forest plot** 85](#_Toc137455708)

[**Figure S28 SV victim; females; funnel plot** 85](#_Toc137455709)

[**Figure S29 SV victim; cross sectional; forest plot** 86](#_Toc137455710)

[**Figure S30 SV victim; cross sectional; funnel plot** 86](#_Toc137455711)

[**Figure S31 SV victim; population-based; forest plot** 87](#_Toc137455712)

[**Figure S32 SV victim; population-based; funnel plot** 87](#_Toc137455713)

[**Figure S33 SV victim; adjusted; forest plot** 88](#_Toc137455714)

[**Figure S34 SV victim; adjusted; funnel plot** 88](#_Toc137455715)

**Table S1 Providers in UNIKA (University of Navarra bibliographic aggregator) with at least 10 results**

| **Bibliographic Provider** |
| --- |
| Academic Search Index |
| Airiti Library eBooks & Journals - 華藝線上圖書館 |
| APA PsycArticles |
| APA PsycInfo |
| BASE |
| BiblioBoard |
| Bibliotheksverbund Bayern |
| Biomedical Index |
| Books at JSTOR |
| British Library Document Supply Centre Inside Serials & Conference Proceedings |
| British Library EThOS |
| Business Insights Global |
| Business Insights: Essentials |
| Business Source Ultimate |
| Cambridge Core Books |
| Catálogo de la Biblioteca de la Universidad de Navarra |
| China Science & Technology Journal Database |
| CINAHL with Full Text |
| ClinicalTrials.gov |
| Complementary Index |
| Credo Reference |
| Dialnet Plus |
| Directory of Open Access Journals |
| DynaMed |
| eArticle |
| eBook Academic Collection (EBSCOhost) |
| eBook Collection (EBSCOhost) |
| eBook Index |
| ELibrary.RU |
| ERIC |
| eScholarship |
| FRANCIS Archive |
| Fuente Académica Premier |
| Gale Academic OneFile |
| Gale Academic OneFile Select |
| Gale General OneFile |
| Gale Health and Wellness |
| Gale In Context: Canada |
| Gale in Context: College |
| Gale in Context: Environmental Studies |
| Gale In Context: Global Issues |
| Gale In Context: High School |
| Gale In Context: Opposing Viewpoints |
| Gale In Context: Science |
| Gale Literature: Book Review Index |
| Gale OneFile: Business |
| Gale OneFile: CPI.Q |
| Gale OneFile: Health and Medicine |
| Gale OneFile: High School Edition |
| Gale OneFile: News |
| Harvard Library Bibliographic Dataset |
| Idealonline |
| Journals@OVID |
| JSTOR Journals |
| KERIS Theses & Dissertations |
| Korean Studies Information Service System |
| Medical Online-E |
| MEDLINE |
| NARCIS |
| Networked Digital Library of Theses & Dissertations |
| NewsBank |
| NORA (Norwegian Open Research Archive) |
| OAIster |
| OpenAIRE |
| OpenDissertations |
| PASCAL Archive |
| Psychology and Behavioral Sciences Collection |
| Regional Business News |
| Research Starters |
| SAGE Knowledge |
| Science Citation Index Expanded |
| ScienceDirect |
| Scopus® |
| Social Sciences Citation Index |
| Springer Nature eBooks |
| Springer Nature Journals |
| Supplemental Index |
| SveMed+ |
| SwePub |
| TDX |
| USPTO Patent Applications |
| VLeBooks |
| 스콜라(Scholar) |

**Table S2 Search strategy**

(ADHD OR adhd OR attention deficit disorder with hyperactivity OR syndrome hyperkinetic OR hyperkinetic syndrome OR hyperactivity disorder OR hyperactive child syndrome OR childhood hyperkinetic syndrome OR attention deficit hyperactivity disorders OR attention deficit hyperactivity disorder OR adhd attention deficit hyperactivity disorder OR adhd OR overactive child syndrome OR attention deficit hyperkinetic disorder OR hyperkinetic disorder OR attention deficit disorder hyperactivity OR attention deficit disorders hyperactivity OR child attention deficit disorder OR hyperkinetic syndromes OR syndromes hyperkinetic OR hyperkinetic syndrome childhood)

AND

((sex offenses[MeSH Terms] OR rape OR ((sexual OR sex OR gender-based OR gender-related) AND (violence OR assault OR assaults OR offense OR offenses OR abuse OR abuses))) OR (Intimate partner violence OR Spouse abuse OR Physical Abuse OR Domestic violence OR Gender-Based Violence)

OR

(( Abused OR Abusing OR Abusive OR Aggressive OR Battered OR Batterer OR Battering OR Bullied OR Bullying OR Coerced OR coercive OR Harassed OR Harasser OR Maltreated OR Maltreater OR Neglected OR Violent OR Batter OR Coerce OR Harass OR Maltreat OR Neglect OR Victimize OR Victim OR Abuse OR Aggression OR Assault OR Bully OR Coercion OR Harassment OR Ill-treatment OR Maltreatment OR Mistreatment OR Victimization OR Victimizing OR Violence ) AND ( Conjugal OR Dating OR Domestic OR Marital OR Matrimonial OR Nuptial OR Spousal OR Companion OR Consort OR Couple OR Date OR Husband OR Marriage OR Partner OR Relation OR Relationship OR Significant other OR Spouse OR Wife OR Companions OR Consorts OR Couples OR Husbands OR Marriages OR Partners OR Relations OR Relationships OR Significant others OR Spouses OR Wives )))

**Table S3 Operationalization of the Newcastle-Ottawa scale for case-control studies**

| **Selection** |
| --- |
| 1) Is the case definition adequate? a) yes, with independent validation OR RECORD LINKAGE* b) based on self reports c) no description |
| 2) Representativeness of the cases a) consecutive or obviously representative series of cases* b) potential for selection biases or not stated |
| 3) Selection of Controls a) community controls* b) hospital controls c) no description |
| 4) Definition of Controls a) no history of IPV* b) no description of source |
| **Comparability** (up to two stars) |
| 1) Comparability of cases and controls on the basis of the design or analysis a) study controls for SEX* b) study controls for AGE* |
| **Exposure** |
| 1) Ascertainment of exposure a) secure record (e.g. surgical records/RECORD LINKAGE)* b) structured interview where blind to case/control status* c) interview not blinded to case/control status d) written self report or medical record only e) no description |
| 2) Same method of ascertainment for cases and controls a) yes* b) no |
| 3) Non-Response rate a) same rate for both groups* b) non respondents described c) rate different and no designation |

**Table S4 Operationalization of the Newcastle-Ottawa scale for cohort studies**

| **Selection** |
| --- |
| 1) Representativeness of the exposed cohort a) truly representative of the average PERSON WITH ADHD in the community*  b) somewhat representative of the average PERSON WITH ADHD in the community* c) selected group of users e.g. nurses, volunteers d) no description of the derivation of the cohort |
| 2) Selection of the non exposed cohort a) drawn from the same community as the exposed cohort* b) drawn from a different source c) no description of the derivation of the non exposed cohort |
| 3) Ascertainment of exposure a) secure record (RECORD LINKAGE)* b) structured interview* c) written self report d) no description |
| 4) Demonstration that outcome of interest was not present at start of study a) yes* b) no |
| **Comparability** (up to two stars) |
| 1) Comparability of cohorts on the basis of the design or analysis a) study controls for SEX* b) study controls for AGE* |
| **Outcome** |
| 1) Assessment of outcome  a) independent blind assessment*  b) record linkage* c) self report  d) no description |
| 2) Was follow-up long enough for outcomes to occur a) yes (5 YEARS)* b) no |
| 3) Adequacy of follow up of cohorts a) complete follow up - all subjects accounted for*  b) subjects lost to follow up unlikely to introduce bias - small number lost - > 20 % follow up, or description provided of those lost)* c) follow up rate <20% and no description of those lost d) no statement |

**Table S5 Operationalization of the Newcastle-Ottawa scale for cross-sectional studies**

| **Selection** |
| --- |
| Representativeness of the sample: a) Truly representative of the average in the target population* (all subjects or random sampling) b) Somewhat representative of the average in the target population* (non-random sampling) c) Selected group of users (THIS INCLUDES SELECTION OF EXPOSED VS UNEXPOSED) d) No description of the sampling strategy |
| 2) Sample size: a) Justified and satisfactory*  b) Not justified |
| 3) Non-respondents: a) Comparability between respondents and non-respondents characteristics is established, and the response rate is satisfactory* b) The response rate is unsatisfactory, or the comparability between respondents and non-respondents is unsatisfactory c) No description of the response rate or the characteristics of the responders and the non-responders |
| 4) Ascertainment of the exposure (risk factor): a) Validated measurement tool: CLINICAL DIAGNOSIS** b) Non-validated measurement tool, but the tool is available or described. RECORD LINKAGE*  c) No description of the measurement tool. SELF REPORT OR QUESTIONNAIRE |
| **Comparability** (up to two stars) |
| 1) The subjects in different outcome groups are comparable, based on the study design or analysis. Confounding factors are controlled  a) The study controls for AGE* b) The study controls for SEX* |
| **Outcome** |
| 1) Assessment of the outcome: (up to two stars) a) Independent blind assessment OR Record linkage** b) VALIDATED QUESTIONNAIRE* c) Self report/NON-VALIDATED QUESTIONNAIRE. d) No description |
| 2) Statistical test: a) The statistical test used to analyze the data is clearly described and appropriate, and the measurement of the association is presented, including confidence intervals and the probability level (p value) OR ALL NUMBERS OF THE 2X2 MATRIX ARE PRESENTED (CALCULATION BY PERCENTAGES DOES NOT COUNT)* b) The statistical test is not appropriate, not described or incomplete |

**Table S6 List of planned subgroup meta-analyses and whether they were finally carried out**

| **Subanalysis** | **IPV victim** | **IPV perpetrator** | **SV victim** | **SV perpetrator** |
| --- | --- | --- | --- | --- |
| **Studies that control for sex either by design or statistically** | **No** | **No** | **No** | **No** |
| **Studies on males** | **No** | **No** | **No** | **No** |
| **Studies on females** | **No** | **No** | **Yes** | **No** |
| **Physical IPV** | **Yes** | **Yes** | **-** | **-** |
| **Psychological IPV** | **No** | **No** | **-** | **-** |
| **Sexual IPV** | **No** | **No** | **-** | **-** |
| **Adolescents** | **No** | **No** | **No** | **No** |
| **Adults** | **No** | **No** | **No** | **No** |
| **Cohort studies** | **No** | **No** | **No** | **No** |
| **Case-control studies** | **No** | **No** | **No** | **No** |
| **Cross-sectional studies** | **No** | **Yes** | **Yes** | **No** |
| **In clinical samples** | **No** | **No** | **No** | **No** |
| **In population-based samples** | **No** | **Yes** | **Yes** | **No** |
| **ADHD diagnosis confirmed clinically or through an administrative register** | **No** | **Yes** | **No** | **No** |
| **Adjusted ORs** | **Yes** | **Yes** | **Yes** | **Yes** |

**Table S7 References excluded after full-text evaluation with reasons for exclusion**

| **Author year** | **Title** | **Journal** | **VOLUME** | **PAGES** | **Reason for exclusion** |
| --- | --- | --- | --- | --- | --- |
|  | Girls with ADHD at higher risk of mental health problems: study |  |  |  | Not empirical |
|  | Findings in attention deficit hyperactivity disorders reported from university of California (childhood attention-deficit/hyperactivity disorder predicts intimate partner victimization in young women) |  |  |  | Not empirical |
|  | An attention deficit hyperactivity disorder diagnosis puts girls at much higher risk for other mental health problems |  |  |  | Not empirical |
|  | Study finds ADHD can hit girls hard |  |  |  | Not empirical |
|  | New attention disorders study findings recently were reported by researchers at Ohio university (childhood ADHD potentiates the association between problematic drinking and intimate partner violence) |  |  |  | Not empirical |
|  | Reports from Ohio university advance knowledge in attention deficit hyperactivity disorders (ADHD symptoms as risk factors for intimate partner violence perpetration and victimization) |  |  |  | Not empirical |
|  | Studies from Ohio university yield new data on attention disorders (rates of intimate partner violence perpetration and victimization among adults with ADHD) |  |  |  | Not empirical |
|  | ADHD girls exhibit increased overt and relational aggression | ADHD Report | 12 | 12-dic | Not empirical |
|  | An exploration of verbal aggressiveness, conflict strategies, and attention deficit hyperactivity disorder (ad/hd) characteristics | Conference Papers -- International Communication Association |  | ene-31 | Not on violence |
|  | Cinsel istismara uğrayan çocuk ve ergenlerin sosyodemografik ve klinik özelliklerinin değerlendirilmesi, psikopatoloji ve ilişkili risk etkenleri / evaluatingsociodemographic and clinical properties of sexually abused children and adolescents, psychopatology and associated risk factors | Çocuk Ve Gençlik Ruh Sağlığı Dergisi | 24 | 155-163 | Inadequate comparator |
|  | Findings from n.jl. Buitelaar et al has provided new information about attention disorders (the impact of ADHD treatment on intimate partner violence in a forensic psychiatry setting) |  |  |  | Not empirical |
|  | Developmental relations between ADHD symptoms and bullying perpetration and victimisation in adolescence (updated may 16, 2020) |  |  |  | Not on violence |
|  | Reports outline psychiatry research from Ain Shams university (aggression in ADHD: relation to salivary cortisol) |  |  |  | Not empirical |
|  | New attention disorders findings from Ohio university reported (examining link between childhood ADHD and sexual assault victimization) | Newspaper Article |  |  | Not empirical |
|  | Findings from university of west Florida update knowledge of violence against women (the link between ADHD and the risk of sexual victimization among college women: expanding the lifestyles/routine activities framework) | Women's Health Weekly | 119-119 | Not empirical |  |
| Addy 2021 | Mental health difficulties, coping mechanisms and support systems among school-going adolescents in Ghana: a mixed-methods study | Plos One | 16 |  | Not on violence |
| Agergård Kareliusson 2017 | Ungdomar med ADHD – välmående, relationer och missbruk |  |  |  | Not on violence |
| Aguado-Gracia 2021 | Lifetime victimization in children and adolescents with ADHD | Journal of Interpersonal Violence | 36 | NP3241-NP3262 | Inadequate comparator |
| Ahmad 2017 | Attention-deficit/hyperactivity disorder, trait impulsivity, and externalizing behavior in a longitudinal sample | Journal of Abnormal Child Psychology | 45 |  | Not on violence |
| Al Ansari 2017 | Outcomes of children with attention deficit/hyperactivity disorder: global functioning and symptoms persistence | Eastern Mediterranean Health Journal | 23 | 589-589 | Not on violence |
| Babinski 1995 | Childhood ADHD and aggression as predictors of later criminal activity: a longitudinal study of risk and protective factors from early childhood through young adulthood |  |  |  | Not on violence |
| Babinski 1999 | Childhood conduct problems, hyperactivity-impulsivity, and inattention as predictors of adult criminal activity | Journal of Child Psychology and Psychiatry and Allied Disciplines | 40 | 347-355 | Not on violence |
| Babinski 2011 | Late adolescent and young adult outcomes of girls diagnosed with ADHD in childhood: an exploratory investigation | Journal of Attention Disorders | 15 | 204-214 | Not on violence |
| Babinski 2019 | Borderline personality features mediate the association between ADHD, odd, and relational and physical aggression in girls | Journal of Attention Disorders | 23 | 838-848 | Not on violence |
| Babinski 2020 | Lifetime caregiver strain among mothers of adolescents and young adults with attention-deficit/hyperactivity disorder | Journal of Family Psychology | 34 | 342-352 | Not on violence |
| Bagwell 2001 | Attention-deficit hyperactivity disorder and problems in peer relations: predictions from childhood to adolescence | Journal of the American Academy of Child and Adolescent Psychiatry | 40 | 1285-1292 | Not on violence |
| Bailey 1997 | Domestic violence and mental illness |  |  |  | Not empirical |
| Balvers 2019 | The impact of parenting on the relationship between ADHD symptoms and aggression in girls |  |  |  | Not on violence |
| Barcai 1974 | A precursor of delinquency: the hyperkinetic disorder of childhood | The Psychiatric Quarterly | 48 | 387-399 | Not empirical |
| Barnow 2001 | Influence of punishment, emotional rejection, child abuse, and broken home on aggression in adolescence: an examination of aggressive adolescents in Germany | Psychopathology | 34 | 167-173 | Not on violence |
| Bebbington 2021 | The mental health of ex-prisoners: analysis of the 2014 English national survey of psychiatric morbidity | Social Psychiatry and Psychiatric Epidemiology |  |  | Not on violence |
| Becker 2017 | Rates of peer victimization in young adolescents with ADHD and associations with internalizing symptoms and self-esteem | European Child & Adolescent Psychiatry | 26 | 201-214 | Not on violence |
| Beckman 2016 | Associations between neurodevelopmental disorders and factors related to school, health, and social interaction in schoolchildren: results from a Swedish population-based survey | Disability and Health Journal | 9 | 663-672 | Not on violence |
| Bell 2021 | Attention-deficit/hyperactivity disorder symptoms and externalizing progression in the lams study: a test of trait impulsivity theory | Journal of the American Academy of Child and Adolescent Psychiatry |  |  | Not on violence |
| Ben-Naim 2017 | Life with a partner with ADHD: the moderating role of intimacy | Journal of Child and Family Studies | 26 | 1365-1373 | Not on violence |
| Bernat 2012 | Risk and direct protective factors for youth violence: results from the national longitudinal study of adolescent health | American Journal of Preventive Medicine | 43 | S57-66 | Not on violence |
| Bettger 2010 | Comparing risk factors for sexual offending between adolescent non-sexual offenders and sexual offenders: a case for improved preventative sexual education | Thesis |  |  | Not on ADHD |
| Biederman 2010 | Adult psychiatric outcomes of girls with attention deficit hyperactivity disorder: 11-year follow-up in a longitudinal case-control study | The American Journal of Psychiatry | 167 | 409-417 | Not on violence |
| Bird 2006 | A study of disruptive behavior disorders in puerto rican youth: ii. Baseline prevalence, comorbidity, and correlates in two sites | Journal of the American Academy of Child and Adolescent Psychiatry | 45 | 1042-1053 | Not on violence |
| Blachman 2005 | Predictors of peer rejection, acceptance, and victimization among girls with and without ADHD |  |  |  | Not on violence |
| Bonomi 2018 | Sexual violence and intimate partner violence in college women with a mental health and/or behavior disability | Journal of Women's Health | 27 | 359-368 | Inadequate comparator |
| Boonmann 2015 | Mental disorders in juveniles who sexually offended: a meta-analysis | Aggression and Violent Behavior |  | 241-249 | Systematic review of interest |
| Booth 2014 | Mental illness and sexual offending | Psychiatric Clinics of North America |  | 183-+ | Not empirical |
| Brinig 2012 | Explaining abuse of the disabled child | Family Law Quarterly | 46 | 269-296 | Not on ADHD |
| Buitelaar 2014 | Impact of treatment of ADHD on intimate partner violence (ITAP), a study protocol | BMC Psychiatry | 14 | 336-336 | Not empirical |
| Buitelaar 2016 | Screening for ADHD among offenders of intimate partner violence | International Journal of Forensic Mental Health | 15 | 256-264 | Inadequate comparator |
| Buitelaar 2020 | ADHD and intimate partner violence. Impact of ADHD as a risk and a treatment factor in intimate partner violence |  |  |  | Systematic review of interest |
| Buitelaar 2020 | ADHD in childhood and/or adulthood as a risk factor for domestic violence or intimate partner violence: a systematic review | Journal of Attention Disorders | 24 | 1203-1214 | Systematic review of interest |
| Buitelaar 2020 | Type and severity of intimate partner violence in offenders with and without ADHD |  |  |  | Inadequate comparator |
| Buitelaar 2021 | The impact of ADHD treatment on intimate partner violence in a forensic psychiatry setting | Journal of Attention Disorders | 25 | 1021-1031 | Inadequate comparator |
| Butler 2013 | Child sexual assault: risk factors for girls | Child Abuse & Neglect |  | 643-652 | Not on ADHD |
| Byrne 2015 | Adult attention deficit disorder and aggressive behaviour: an exploration of relationships between brown attention-deficit disorder scales and the aggression questionnaire | Psychiatry, Psychology and Law | 22 | 407-416 | Not on violence |
| Cackowski 2017 | Anger and aggression in borderline personality disorder and attention deficit hyperactivity disorder - does stress matter? | Borderline Personality Disorder and Emotion Dysregulation | 4 | 06-jun | Not on violence |
| Capaldi 2016 | Adolescent/young adult romantic relationships and psychopathology |  |  |  | Not empirical |
| Capusan 2017 | Childhood maltreatment and adult attention deficit hyperactivity disorder symptoms: a twin study | European Neuropsychopharmacology | 27 MA - 1 | S139-S139 | Not on violence |
| Capuzzi 2022 | Screening for ADHD symptoms among criminal offenders: exploring the association with clinical features | Healthcare |  |  | Not on violence |
| Carabellese 2016 | [ADHD and illegal conduct: a survey in juvenile justice services in Puglia] | Rivista di Psichiatria | 51 | 156-163 | Not on violence |
| Carpentier 2011 | Correlates of recidivism among adolescents who have sexually offended | Sexual Abuse |  | 434-455 | Inadequate comparator |
| Caspers 2020 | The Iowa adoption studies, 1975-2008 |  |  |  | Not on violence |
| Chang 2015 | Psychiatric disorders and violent reoffending: a national cohort study of convicted prisoners in Sweden | Lancet Psychiatry | 2 | 891-900 | Not on violence |
| Chou 2014 | Bullying victimization and perpetration and their correlates in adolescents clinically diagnosed with ADHD | Journal of Attention Disorders | 22 | 25-34 | Not on violence |
| Christoffersen 2019 | Violent crime against children with disabilities: a nationwide prospective birth cohort-study | Child Abuse & Neglect | 98 | 104150-104150 | Not on violence |
| Christoffersen 2020 | Sexual crime against schoolchildren with disabilities: a nationwide prospective birth cohort study | Journal of Interpersonal Violence |  | 886260520934442-886260520934442 | Not on violence |
| Cohen 2002 | Sexuality for women with AD/HD | Book Section |  |  | Not empirical |
| Colins 2010 | Psychiatric disorders in detained male adolescents: a systematic literature review | The Canadian Journal of Psychiatry | 55 | 255-263 | Systematic review of interest |
| Colins 2011 | Psychiatric Disorder in Detained Male Adolescents as Risk Factor for Serious Recidivism | The Canadian Journal of Psychiatry | 56 | 44-50 | Not on violence |
| Colins 2013 | Mental health problems and recidivism among detained male adolescents from various ethnic origins | European Child & Adolescent Psychiatry | 22 | 481-490 | Not on violence |
| Copeland 2007 | Childhood psychiatric disorders and young adult crime: a prospective, population-based study | American Journal of Psychiatry |  | 1668-1675 | Not on violence |
| Cunningham 2021 | Profiles of juveniles with sex offense charges referred for competence evaluations | Journal of Forensic Sciences | 66 | 1829-1840 | Inadequate comparator |
| Curnoe 2013 | Psychological profile of sex offenders using weapons in their crimes | Journal of Sexual Aggression |  | 55-68 | Inadequate comparator |
| Dåderman 2004 | The prevalence of dyslexia and AD/HD in a sample of forensic psychiatric rapists | Nordic Journal of Psychiatry |  | 371-381 | Inadequate comparator |
| Dåderman 2008 | Lack of psychopathic character (Rorschach) in forensic psychiatric rapists | Nordic Journal of Psychiatry |  | 176-185 | Inadequate comparator |
| De Bellis 2005 | Prefrontal cortex, thalamus, and cerebellar volumes in adolescents and young adults with adolescent-onset alcohol use disorders and comorbid mental disorders | Alcoholism, Clinical and Experimental Research |  | 1590-1600 | Not on violence |
| DePrince 2016 | Preventing revictimization in teen dating relationships, 2010-2013, Denver, Colorado |  |  |  | Inadequate comparator |
| Di Lorenzo 2021 | Children and adolescents with ADHD followed up to adulthood: a systematic review of long-term outcomes | Acta Neuropsychiatrica |  | 42370 | Not on violence |
| Dowdy-Hazlett 2021 | Predictors of mental health diagnoses among youth in psychiatric residential care: a retrospective case record analysis | Child & Adolescent Social Work Journal |  |  | Inadequate comparator |
| Dubowitz 1993 | A follow-up-study of behavior problems associated with child sexual abuse | Child Abuse & Neglect | 17 | 743-754 | Not on violence |
| DuPaul | College students with ADHD: what do we know and where do we go from here? |  |  |  | Not empirical |
| Dupont 2008 | Characteristics and motives of college students who engage in nonmedical use of methylphenidate | The American Journal On Addictions | 17 | 167-171 | Not on ADHD |
| Eastvold 2011 | Executive function profiles of pedophilic and nonpedophilic child molesters | Journal of the International Neuropsychological Society |  | 295-307 | Inadequate comparator |
| Ebejer 2012 | Attention deficit hyperactivity disorder in Australian adults: prevalence, persistence, conduct problems and disadvantage | Plos One | 7 | e47404-e47404 | Not on violence |
| Edberg 2022 | Crimes and sentences in individuals with intellectual disability in a forensic psychiatric context: a register-based study | Epidemiology and Psychiatric Sciences | 31 |  | Not on violence |
| Edinburgh 2006 | Gender differences in extrafamilial sexual abuse experiences among young teens | The Journal of School Nursing : the Official Publication of the National Association of School Nurses | 22 | 278-284 | Inadequate comparator |
| Efron 2021 | Peer victimization in children with ADHD: a community-based longitudinal study | Journal of Attention Disorders | 25 | 291-299 | Not on violence |
| Egle 2004 | Attention-deficit/hyperactivity disorder (ADHD) in adulthood | Psychotherapie Psychosomatik Medizinische Psychologie | 54 | 137-147 | Not on violence |
| Einarsson 2009 | Screening for attention-deficit hyperactivity disorder and co-morbid mental disorders among prison inmates | Nordic Journal of Psychiatry | 63 | 361-367 | Not on violence |
| Ek 2018 | ADHD och kriminalitet - en studie om frivårdens arbete med klienter som har ADHD | Thesis |  |  | Not empirical |
| Eklund 2003 | Childhood behaviour as related to subsequent drinking offences and violent offending: a prospective study of 11- to 14-year-old youths into their fourth decade | Criminal Behaviour and Mental Health | 13 | 294-309 | Not on violence |
| Eklund 2005 | Monoamine oxidase activity and tri-iodothyronine level in violent offenders with early behavioural problems | Neuropsychobiology | 52 | 122-129 | Not on violence |
| Elkins 2007 | Prospective effects of attention-deficit/hyperactivity disorder, conduct disorder, and sex on adolescent substance use and abuse | Archives of General Psychiatry | 64 | 1145-1152 | Not on violence |
| Elkins 2011 | The impact of attention-deficit/hyperactivity disorder on preadolescent adjustment may be greater for girls than for boys | Journal of Clinical Child and Adolescent Psychology | 40 | 532-545 | Not on violence |
| Elliott 1976 | Neurological factors in violent behavior (the dyscontrol syndrome) | The Bulletin of the American Academy of Psychiatry and the Law | 4 | 297-315 | Not on ADHD |
| Elliott 1982 | Neurological findings in adult minimal brain dysfunction and the dyscontrol syndrome | The Journal of Nervous and Mental Disease | 170 | 680-687 | Not on ADHD |
| Elliott 1992 | Violence - the neurologic contribution - an overview | Archives of Neurology |  | 595-603 | Not empirical |
| Eme 2009 | Male life-course persistent antisocial behavior: a review of neurodevelopmental factors | Aggression and Violent Behavior |  | 348-358 | Not empirical |
| Ende 2015 | Impulsivity and aggression in female bpd and ADHD patients: association with ACC glutamate and Gaba concentrations | Neuropsychopharmacology | 41 | 410-418 | Not on violence |
| Engel 2012 | ADHS IN EINER entziehungsmaßregel = ADHD IN FORENSIC PSYCHIATRY FOR WITHDRAWAL AND ABSTINENCE MEASURE | Nervenheilkunde |  | 42-47 | Inadequate comparator |
| Eray 2018 | Assessment of forensic cases attending to a child psychiatry clinic objective |  |  |  | Not on violence |
| Ermer 2008 | Forensic psychiatry and psychology | Zeitschrift Fur Psychiatrie Psychologie Und Psychotherapie | 56 | 75-77 | Not empirical |
| Erskine 2016 | Long-term outcomes of attention-deficit/hyperactivity disorder and conduct disorder: a systematic review and meta-analysis | Journal of the American Academy of Child & Adolescent Psychiatry | 55 | 841-850 | Not on violence |
| Etherington 1993 | Diagnostic and personality differences of juvenile sex offenders, non- sex offenders, and non-offenders | Thesis |  |  | Inadequate comparator |
| Evinç 2014 | Child maltreatment and associated factors among children with ADHD: a comparative study | Turkish Journal of Pediatrics | 56 | nov-22 | Not on violence |
| Faigel 1995 | Attention deficit disorder in college students: facts, fallacies, and treatment | Journal of American College Health | 43 | 147-155 | Not empirical |
| Faigel 1995 | Attention deficit disorder during adolescence: a review | The Journal of Adolescent Health | 16 | 174-184 | Not empirical |
| Faraone 2021 | The world federation of ADHD international consensus statement: 208 evidence-based conclusions about the disorder | Neuroscience and Biobehavioral Reviews | 128 | 789-818 | Not empirical |
| Farrington 1989 | Early predictors of adolescent aggression and adult violence | Violence and Victims | 4 | 79-100 | Not on violence |
| Farrington 2009 | Development of adolescence-limited, late-onset, and persistent offenders from age 8 to age 48 | Aggressive Behavior | 35 | 150-163 | Not on violence |
| Fazel 2008 | Mental disorders among adolescents in juvenile detention and correctional facilities: a systematic review and metaregression analysis of 25 surveys | Journal of the American Academy of Child and Adolescent Psychiatry |  | 1010-1019 | Not on violence |
| Feilhauer 2012 | Differential associations between psychopathy dimensions, types of aggression, and response inhibition | Aggressive Behavior | 38 | 77-88 | Not on ADHD |
| Fergusson 1997 | Attentional difficulties in middle childhood and psychosocial outcomes in young adulthood | Journal of Child Psychology and Psychiatry, and Allied Disciplines | 38 | 633-644 | Not on violence |
| Fernandez 2022 | Associations between omega-3 index, dopaminergic genetic variants and aggressive and metacognitive traits: a study in adult male prisoners | Nutrients |  |  | Not on ADHD |
| Fındıklı 2016 | Suça sürüklenen çocuklarin suç tipleri, sosyodemografik ve klinik özellikleri |  |  |  | Not on ADHD |
| Fischer 1993 | The adolescent outcome of hyperactive children: predictors of psychiatric, academic, social, and emotional adjustment | Journal of the American Academy of Child and Adolescent Psychiatry | 32 | 324-332 | Not on violence |
| Fischer 2002 | Young adult follow-up of hyperactive children: self-reported psychiatric disorders, comorbidity, and the role of childhood conduct problems and teen cd | Journal of Abnormal Child Psychology | 30 | 463-475 | Not on violence |
| Fite 2014 | Further evaluation of associations between attention-deficit/hyperactivity and oppositional defiant disorder symptoms and bullying-victimization in adolescence | Child Psychiatry and Human Development | 45 | 32-41 | Not on violence |
| Fletcher 2009 | Long-term consequences of childhood ADHD on criminal activities | the Journal of Mental Health Policy and Economics | 12 | 119-138 | Not on violence |
| Flory 2006 | Childhood ADHD predicts risky sexual behavior in young adulthood | Journal of Clinical Child and Adolescent Psychology | 35 | 571-577 | Not on violence |
| Flory 2007 | Serotonergic function in children with attention-deficit hyperactivity disorder: relationship to later antisocial personality disorder | The British Journal of Psychiatry | 190 | 410-414 | Not on violence |
| Flynn 2004 | Diagnosis, executive function and coping styles in young offenders |  |  |  | Inadequate comparator |
| Fogleman 2019 | Emotion regulation accounts for the relation between ADHD and peer victimization | Journal of Child and Family Studies | 28 | 2429-2442 | Not on violence |
| Foley 1996 | The relationship of attention deficit hyperactivity disorder and conduct disorder to juvenile delinquency: legal implications | The Bulletin of the American Academy of Psychiatry and the Law | 24 | 333-345 | Not empirical |
| Folino 2011 | Juvenile offenders assessment | Current Opinion In Psychiatry | 24 | 436-441 | Not empirical |
| Franco 2010 | Psychopathological problems in neurological disorders of children and adolescents. Risk factors and prevention |  |  |  | Not empirical |
| Franke 2020 | Special issue on the neurobiology of aggressive behaviour in the context of ADHD and related disorders |  |  |  | Not empirical |
| Frappier 2018 | Sleep problems and interpersonal violence in youth in care under the Quebec child welfare society | Sleep Medicine |  | 52-56 | Not on violence |
| Fridh 2018 | Bullying, violence and mental distress among young people. Cross-sectional population-based studies in Scania, Sweden |  |  |  | Not on ADHD |
| Fritz 2008 | Psychopathy and violence in juvenile delinquents: what are the associated factors? | International Journal of Law and Psychiatry | 31 | 272-279 | Not on ADHD |
| Füessl 2014 | [ADHD, bullying and suicidality in children and adolescents] | MMW Fortschritte Der Medizin | 156 | 40-40 | Not on violence |
| Fuller-Thomson 2016 | Attention-deficit/hyperactivity disorder casts a long shadow: findings from a population-based study of adult women with self-reported ADHD | Child: Care, Health and Development | 42 | 918-927 | Not on violence |
| Galan 2020 | Early childhood trajectories of conduct problems and hyperactivity/attention problems: predicting adolescent and adult antisocial behavior and internalizing problems | Journal of Clinical Child and Adolescent Psychology | 49 | 200-214 | Not on violence |
| Galli 1999 | The psychiatric diagnoses of twenty-two adolescents who have sexually molested other children | Comprehensive Psychiatry | 40 | 85-88 | Inadequate comparator |
| Gannon 2008 | Rape: psychopathology, theory and treatment | Clinical Psychology Review |  | 982-1008 | Not empirical |
| Gau 2001 | Individual and familial correlates and outcomes of attention deficit hyperactivity disorder: a longitudinal follow-up study of a high risk sample (high-risk children) |  |  |  | Not on violence |
| Ghirardi 2021 | Neurodevelopmental disorders and subsequent risk of violent victimization: exploring sex differences and mechanisms | Psychological Medicine |  |  | Not on violence |
| Giotakos 2005 | Aggression, impulsivity, and plasma sex hormone levels in a group of rapists, in relation to their history of childhood attention-deficit/hyperactivity disorder symptoms | Journal of Forensic Psychiatry & Psychology | 16 | 423-433 | Inadequate comparator |
| Goodman 2008 | ADHD and aggression as correlates of suicidal behavior in assaultive prepubertal psychiatric inpatients | Suicide & Life-Threatening Behavior | 38 | 46-59 | Not on violence |
| Gordon 2011 | Relationship between ADHD symptoms and offending behaviour and breaches of prison discipline among incarcerated youths in Scotland | Journal of Epidemiology and Community Health | 65 | A188-A188 | Not on violence |
| Gordon 2012 | Does ADHD matter? Examining attention deficit and hyperactivity disorder on the likelihood of recidivism among detained youth | Journal of Offender Rehabilitation | 51 | 497-518 | Not on violence |
| Gordon 2012 | Exploring the relationship between ADHD symptoms and prison breaches of discipline amongst youths in four Scottish prisons | Public Health | 126 | 343-348 | Not on violence |
| Gotby 2018 | Childhood neurodevelopmental disorders and risk of coercive sexual victimization in childhood and adolescence - a population-based prospective twin study | Journal of Child Psychology and Psychiatry | 59 | 957-965 | Not on violence |
| Greene 1997 | Adolescent outcome of boys with attention-deficit/hyperactivity disorder and social disability: results from a 4-year longitudinal follow-up study | Journal of Consulting and Clinical Psychology | 65 | 758-758 | Not on violence |
| Gretton 2011 | The mental health needs of incarcerated youth in British Columbia, Canada | International Journal of Law and Psychiatry | 34 | 109-115 | Not on violence |
| Gudjonsson 2012 | A national epidemiological study of offending and its relationship with ADHD symptoms and associated risk factors | Journal of Attention Disorders | 18 | mar-13 | Not on violence |
| Guendelman 2016 | Early-adult correlates of maltreatment in girls with attention-deficit/hyperactivity disorder: increased risk for internalizing symptoms and suicidality | Development and Psychopathology | 28 | ene-14 | Not on violence |
| Guerreiro 2011 | [Attention-deficit hyperactivity disorder in adults: implications to forensic medicine] | Acta Medica Portuguesa | 24 | 319-326 | Not empirical |
| Gunter 2006 | Adult outcomes of attention deficit hyperactivity disorder and conduct disorder: are the risks independent or additive? | Annals of Clinical Psychiatry : Official Journal of the American Academy of Clinical Psychiatrists | 18 | 233-237 | Not on violence |
| Gutt 2013 | Crianças e adolescentes em risco para esquizofrenia e transtorno afetivo bipolar: um estudo comparativo |  |  |  | Not on violence |
| Hadianfard 2014 | Child abuse in group of children with attention deficit-hyperactivity disorder in comparison with normal children | International Journal of Community Based Nursing and Midwifery | 2 | 77-84 | Inadequate comparator |
| Halkett | An exploratory investigation of childhood sexual abuse and other theory-driven predictors of sex work among women with and without childhood ADHD | Journal of Child & Adolescent Trauma |  |  | Not on violence |
| Harakeh 2012 | Individual and environmental predictors of health risk behaviours among Dutch adolescents: the HBSC study | Public Health |  |  | Not on violence |
| Harlow 2009 | Review of mental health and violent youth: a developmental/lifecourse perspective | The Journal of Clinical Psychiatry | 70 | 940-941 | Not empirical |
| Harris 1999 | Adult attention deficit disorder and child abuse |  |  |  | Inadequate comparator |
| Hays 2000 | Impulsivity and its relationship to aggression, social cognition, and peer status |  |  |  | Not on violence |
| Hechtman 2016 | Functional adult outcomes 16 years after childhood diagnosis of attention-deficit/hyperactivity disorder: MTA results | Journal of the American Academy of Child and Adolescent Psychiatry |  | 945-952.e2 | Not on violence |
| Heiman 2015 | Cyberbullying involvement among students with ADHD: relation to loneliness, self-efficacy and social support | European Journal of Special Needs Education | 30 | 15-29 | Not on violence |
| Helgason 2020 | Behavioral problems associated with an adolescent's ADHD symptoms and the family role as a possible protective factor |  |  |  | Not on violence |
| Hines 2016 | Sexual aggression experiences among male victims of physical partner violence: prevalence, severity, and health correlates for male victims and their children | Archives of Sexual Behavior | 45 | 1133-1151 | Not on ADHD |
| Hofvander 2010 | Fc02-01 - trait aggression in adult psychiatry is predicted by childhood hyperactivity, conduct disorder, adult substance abuse, and low cooperativeness | European Psychiatry | 25 | 181-181 | Not on violence |
| Hofvander 2011 | Life history of aggression scores are predicted by childhood hyperactivity, conduct disorder, adult substance abuse, and low cooperativeness in adult psychiatric patients | Psychiatry Research | 185 | 280-285 | Not on violence |
| Holloway 2019 | Sexual assault among college students with disabilities: the hidden victims | Human Development and Family Sciences Undergraduate Honors Theses |  |  | Inadequate comparator |
| Holloway 2022 | Experiences of sexual assault and rape among college students with disabilities | Journal of American College Health |  |  | nadequate comparator |
| Honrath 2021 | Neuropsychologische und psychiatrische einflussfaktoren (auto-) aggressiven verhaltens: eine evaluation großer kohortenstudien zu der huntington krankheit und jugendlichen mit externalisierendem verhalten |  |  |  | Not on ADHD |
| Hoshino 2002 | Use experience of the ann mud course for the battered adolescents who repeated sexual crimes such as i-e-16 paedophilia, the fetishism | Japanese Bulletin of Social Psychiatry |  | 141-141 | Not on ADHD |
| Houtepen 2019 | Social support, attachment and externalizing behavior in forensic patients with attention-deficit hyperactivity disorder | International Journal of Law and Psychiatry | 64 | 106-116 | Not on violence |
| Huang 2017 | ADHD and adverse childhood experiences |  |  |  | Not on violence |
| Isaksson 2018 | Original article: the danger of being inattentive – ADHD symptoms and risky sexual behaviour in Russian adolescents | European Psychiatry | 47 | 42-48 | Not on violence |
| İzmir 2019 | Internet use and aggression in adolescents with attention deficit hyperactivity disorder | Dusunen Adam-Journal of Psychiatry and Neurological Sciences | 32 | 185-193 | Not on violence |
| Jaisoorya 2020 | Prevalence and correlates of self-reported ADHD symptoms in children attending school in India | Journal of Attention Disorders | 24 | 1711-1715 | Not on violence |
| Joshi 2009 | Suicidal ideation after supervised visits with biological mom: depressed mood in a child in foster care | Book Section |  |  | Not empirical |
| Kafka 2012 | Axis I psychiatric disorders, paraphilic sexual offending and implications for pharmacological treatment | The Israel Journal of Psychiatry and Related Sciences |  | 255-261 | Not on violence |
| Karl 2016 | Review of the distracted couple: the impact of ADHD on adult relationships | Journal of Sex & Marital Therapy | 42 | 290-292 | Not empirical |
| Karlsson 2021 | ADHD-symtom som prediktor för utsatthet för brott : en kvantitativ enkätundersökning ; symptoms of attention deficit hyperactivity disorder as predictors of victimization : a quantitative survey | Thesis |  |  | Not on violence |
| Kennedy 2017 | 156 childhood ADHD as a risk factor for violence victimisation in adulthood | Injury Prevention | 23 | A58-A58 | Not on violence |
| Kessler 2014 | National comorbidity survey: reinterview (ncs-2), 2001-2002 |  |  |  | Not empirical |
| Kilcarr 2002 | 12 - making marriages work for individuals with ADHD |  |  |  | Not empirical |
| Klomek 2016 | Victimization by bullying and attachment to parents and teachers among student who report learning disorders and/or attention deficit hyperactivity disorder | Learning Disability Quarterly | 39 | 182-190 | Not on violence |
| Kofler 2015 | Developmental trajectories of aggression, prosocial behavior, and social-cognitive problem solving in emerging adolescents with clinically elevated attention-deficit/hyperactivity disorder symptoms | Journal of Abnormal Psychology | 124 | 1027-1042 | Not on violence |
| Kooij 2018 | Attention-deficit hyperactivity disorder (ADHD), intimate relationships and sexuality |  |  |  | Not empirical |
| Krans 2017 | De ontwikkeling van traumasymptomen bij jongens en meisjes na blootstelling aan partnergeweld |  |  |  | Inadequate comparator |
| Kulesza 2006 | Attention-deficit/hyperactivity disorder and executive functions : potential vulnerabilities for bully/victimization behaviors |  |  |  | Not on violence |
| Kumar Sharma 2014 | Prevalence and psychosocial factors of aggression among youth | Indian Journal of Psychological Medicine | 36 | 48-53 | Not on violence |
| Kurnaz 2013 | An examination of peer aggression and victimization in relation to psycho-social variables. (English) | Akran Saldırganlığı Ve Mağduriyetinin Psiko-Sosyal Değiskenler Açısından Đncelenmesi. (Turkish) | 46 | 317-342 | Not on violence |
| Kurosaki 2013 | Evaluation of characteristics of cognitive and behavioral functions in abused children | Juntendo Medical Journal | 59 | 490-495 | Not on violence |
| Ladd 2006 | Peer rejection, aggressive or withdrawn behavior, and psychological maladjustment from ages 5 to 12: an examination of four predictive models | Child Development | 77 | 822-846 | Not on ADHD |
| Lam 2010 | Early adolescent outcome of attention-deficit hyperactivity disorder in a Chinese population: 5-year follow-up study | Hong Kong Medical Journal | 16 | 257-264 | Not on violence |
| Lambert 1988 | Adolescent outcomes for hyperactive children: perspectives on general and specific patterns of childhood risk for adolescent educational, social, and mental health problems | American Psychologist | 43 | 786-799 | Not on violence |
| Lane 1998 | Cognitive and clinical implications of sexual victimization on boys | Thesis |  |  | Not on violence |
| Langevin 2006 | Generational substance abuse among male sexual offenders and paraphilics | Victims & Offenders | 1 | 395-409 | Not on ADHD |
| Langevin 2010 | A comparison of psychopathy, attention deficit hyperactivity disorder, and brain dysfunction among sex offenders | Journal of Forensic Psychology Practice | 10 | 177-200 | Not on violence |
| Langevin 2011 | Psychopathy, ADHD, and brain dysfunction as predictors of lifetime recidivism among sex offenders | International Journal of Offender Therapy & Comparative Criminology |  |  | Inadequate comparator |
| Langley 2010 | Adolescent clinical outcomes for young people with attention-deficit hyperactivity disorder | The British Journal of Psychiatry : the Journal of Mental Science | 196 | 235-240 | Not on violence |
| Latvala 2022 | Association of intellectual disability with violent and sexual crime and victimization: a population-based cohort study | Psychological Medicine |  |  | Inadequate comparator |
| Lazaratou 2012 | [Attention-deficit hyperactivity disorder or bipolar disorder in childhood?] | Psychiatrike | 23 | 304-313 | Not empirical |
| Lee 2005 | Behavioral and sociometric predictors of adolescent outcomes among girls with and without attention-deficit/hyperactivity disorder (ADHD) |  |  |  | Not on violence |
| Leenarts 2013 | Associations between trauma history and juvenile sexual offending | Journal of Trauma & Treatment | S4 |  | Inadequate comparator |
| Lees 2015 | Could there be a link between ADHD and child sexual abuse? |  |  |  | Not on violence |
| Lewis 2015 | The association between youth violence exposure and attention-deficit/hyperactivity disorder (ADHD) symptoms in a sample of fifth-graders | American Journal of Orthopsychiatry | 85 | 504-513 | Not on ADHD |
| Lie 1992 | Follow-ups of children with attention deficit hyperactivity disorder (ADHD). Review of literature | Acta Psychiatrica Scandinavica | 368 | ene-40 | Not empirical |
| Lindblad 2011 | Sexual abuse allegations by children with neuropsychiatric disorders | Journal of Child Sexual Abuse | 20 | 182-195 | Not empirical |
| Loney 1997 | Associations between clinic-referred boys and their fathers on childhood inattention-overactivity and aggression dimensions | Journal of Abnormal Child Psychology | 25 | 499-509 | Not on violence |
| Løvstad 2012 | An inattentive type. Adults with ADHD. A literature review |  |  |  | Not empirical |
| Lundström 2013 | Childhood neurodevelopmental disorders and violent criminality : a sibling control study | Journal of Autism and Developmental Disorders | 44 | 2707-2716 | Not on violence |
| Machado 2017 | Association between ADHD and psychopathology among prison inmates | European Psychiatry | 41 | S263-S263 | Not on violence |
| Mandell 1998 | An investigation of the presence of adult attention deficit hyperactivity disorder behavior in a population of court mandated domestic violence perpetrators | Thesis |  |  | Inadequate comparator |
| Mannuzza 2008 | Lifetime criminality among boys with attention deficit hyperactivity disorder: a prospective follow-up study into adulthood using official arrest records | Psychiatry Research | 160 | 237-246 | No association data |
| Mariano 2005 | Similarities in aggression, inattention/hyperactivity, depression, and anxiety in middle childhood friendships | Journal of Social and Clinical Psychology | 24 | 471-496 | Not on violence |
| Marks 2000 | Predictors of physical aggression in children with attention-deficit/hyperactivity disorder | CNS Spectrums | 5 | 52-57 | Not on violence |
| Marques 2019 | Crenças, atitudes e experiências de violência no namoro: estudo de uma população clínica de adolescentes |  |  |  | Inadequate comparator |
| Matsuura 2010 | The characteristics of AD/HD symptoms, self-esteem, and aggression among serious juvenile offenders in japan | Research In Developmental Disabilities |  | 1197-1203 | Not on violence |
| Mayne 2012 | The relationship between attention-deficit hyperactivity disorder and adverse experiences in childhood |  |  |  | Inadequate comparator |
| McCall 2015 | ADHD and its role in juvenile sex offending: factors associated with deviant and nondeviant sexual offenses |  |  |  | Inadequate comparator |
| McCarthy | Psychiatric and behavioral characteristics among male adolescents with sexually acting out behaviors admitted to a residential treatment facility | Journal of Psychosocial Nursing and Mental Health Services |  |  | Inadequate comparator |
| McClelland 2016 | Disruptive behavior and parenting in emerging adulthood: mediational effect of parental psychopathology | Journal of Child and Family Studies | 25 | 212-223 | Not on violence |
| McCormick 2017 | The role of mental health and specific responsivity in juvenile justice rehabilitation | Law and Human Behavior | 41 | 55-67 | Not on ADHD |
| McCoy 2019 | Prevalence and clinical correlates of co-occurring attention deficit/hyperactivity disorder and posttraumatic stress disorder in a sample of inpatients being treated for substance use disorder |  |  |  | Not on violence |
| McDonald 2020 | When trauma mimics ADHD |  |  |  | Not empirical |
| McQuade 2017 | Peer victimization and changes in physical and relational aggression: the moderating role of executive functioning abilities | Aggressive Behavior | 43 | 503-512 | Not on violence |
| McQuade 2021 | Childhood ADHD symptoms, parent emotion socialization, and adolescent peer problems: indirect effects through emotion dysregulation | Journal of Youth and Adolescence | 50 | 2519-2532 | Not on violence |
| Mendes 2018 | Habilidade motora, funções executivas e psicopatologia na infância e adolescência |  |  |  | Not on violence |
| Mersch 2016 | Arrest or hospitalization? An examination of the relationship between psychiatric symptoms, traumatic childhood experiences, and socio-ecological factors in forensic mental health system responses to offender behavior | Undergraduate Honors Theses |  |  | Not on violence |
| Mintah 2021 | Executive functioning and theory of mind: links with emerging adult peer, occupational, and romantic adjustment | Thesis |  |  | Not on violence |
| Mohr-Jensen 2016 | A meta-analysis and systematic review of the risks associated with childhood attention-deficit hyperactivity disorder on long-term outcome of arrests, convictions, and incarcerations | Clinical Psychology Review | 48 | 32-42 | Not empirical |
| Molero 2015 | Selective serotonin reuptake inhibitors and violent crime: a cohort study | Plos Medicine | 12 |  | Not on ADHD |
| Morris 2007 | Male juvenile sex offenders: connecting ADHD/add and impulsivity of victim choice | Conference Papers -- American Society of Criminology |  |  | Inadequate comparator |
| Mulsow 2001 | Adult attention deficit hyperactivity disorder, the family, and child maltreatment | Trauma, Violence & Abuse | 2 | 36-50 | Not empirical |
| Murphy 1996 | Attention deficit hyperactivity disorder adults: comorbidities and adaptive impairments | Comprehensive Psychiatry | 37 | 393-401 | Not on violence |
| Murray 2018 | Testing the exacerbation and attenuation hypotheses of the role of anxiety in the relation between ADHD and reactive/proactive aggression: a 10-year longitudinal study | Psychiatry Research | 269 | 585-592 | Not on violence |
| Murray-Close 2010 | Proactive, reactive, and romantic relational aggression in adulthood: measurement, predictive validity, gender differences, and association with intermittent explosive disorder | Journal of Psychiatric Research | 44 | 393-404 | Not on ADHD |
| Nelson 2019 | Children's inattention and hyperactivity, mother's parenting, and risk behaviors in adolescence: a 10-year longitudinal study of Chilean children | Journal of Developmental and Behavioral Pediatrics | 40 | 249-256 | Not on violence |
| Nilsson 2016 | Aggressive antisocial behaviors are related to character maturity in young Swedish violent offenders independent of ADHD | Frontiers In Psychiatry | 7 | 185-185 | Inadequate comparator |
| Nivoli 2017 | Adult attention deficit and hyperactivity disorder in violent prisoners | European Neuropsychopharmacology | 27 MA - P.8.b.012 | S1127-S1127 | Not on violence |
| Nylander 2015 | Risky sexual behaviour among adolescents may be related to ADHD | Acta Paediatrica | 104 | e235-e235 | Not empirical |
| Ok 2018 | Connections of marital satisfaction with mental health symptoms, personality and religion among Muslims |  |  |  | Not on violence |
| O'Leary 2002 | Challenges in a 30-year program of research: conduct disorders and attention deficit hyperactivity disorder, the marital discord and depression link, and partner abuse |  |  |  | Not empirical |
| Örengül 2021 | Peer victimization in preadolescent children with ADHD in turkey | Journal of Interpersonal Violence | 36 | NP6624-NP6642 | Not on violence |
| Ortiz León 2016 | Attention deficit disorder in adulthood and college students |  |  |  | Not empirical |
| Oshaughnessy 1992 | Clinical aspects of forensic assessment of juvenile-offenders | Psychiatric Clinics of North America |  | 721-735 | Not empirical |
| Ouyang 2008 | Attention-deficit/hyperactivity disorder symptoms and child maltreatment: a population-based study | The Journal of Pediatrics | 153 | 851-856 | Not on violence |
| ÖZmen 2016 | The relationship between aggression, empathy skills and serum oxytocin levels in male children and adolescents with attention deficit and hyperactivity disorder | Behavioural Pharmacology | 27 | 681-688 | Not on violence |
| Pallanti 2020 | Adult ADHD in trauma- and stressor-related disorders |  |  |  | Not empirical |
| Parrott 2017 | Deconstructing the associations between executive functioning, problematic alcohol use and intimate partner aggression: a dyadic analysis | Drug and Alcohol Review | 36 | 88-96 | Not on ADHD |
| Pelham 1984 | Peer relations in children with hyperactivity/attention deficit disorder | Journal of Learning Disabilities | 17 | 560-567 | Not empirical |
| Pintado Alcázar 2019 | Estudio empírico sobre responsabilidad penal y TDAH en Italia | Revista Internacional De Doctrina Y Jurisprudencia |  |  | Not empirical |
| Pitcairn 2006 | Extent of victimization and selected psychological, behavioral, and substance use characteristics of Latino adolescents involved in the juvenile justice system |  |  |  | Not on ADHD |
| Pittman 2011 | ADHD impulsivity, discipline referrals, sexual harassment, and criminal sexual deviancy. (cover story) | ADHD Report | 19 | 01-jun | Not empirical |
| Prayez 2012 | [Attention-deficit/hyperactivity disorder (ADHD) and child maltreatment: a review] | Revue Medicale De Bruxelles | 33 | 75-86 | Not empirical |
| Prentky 2006 | Risk management of sexually abusive youth in Massachusetts, 1998-2004 |  |  |  | Not empirical |
| Prinz 1981 | Hyperactive and aggressive behaviors in childhood: intertwined dimensions | Journal of Abnormal Child Psychology | 9 | 191-202 | Not on violence |
| Puzzo 2019 | Attention problems predict risk of violence and rehabilitative engagement in mentally disordered offenders | Frontiers In Psychiatry | 10 |  | Not on ADHD |
| Qadeer 2017 | Polymorphisms in dopaminergic system genes; association with criminal behavior and self-reported aggression in violent prison inmates from Pakistan | Plos One | 12 |  | Not on ADHD |
| Qureshi 1997 | Domestic violence and psychopathology |  |  |  | Not on violence |
| Ramsey 2019 | Elevated ADHD symptoms as a predictor of rule violations among male juvenile offenders | Master's Theses |  |  | Not on ADHD |
| Rangel Araiza 2014 | El trastorno por déficit de atención con y sin hiperactividad (TDA/H) y la violencia: revisión de la bibliografía / Attention deficit disorder with and without hyperactivity (ADHD) and violence: literature review | Salud Mental | 37 | 75-82 | Not empirical |
| Renehan 2022 | Domestic violence perpetrator programmes and neurodiversity |  |  |  | Not empirical |
| Research 2019 | Adult psychiatric morbidity survey, 2014: special licence access |  |  |  | Not on violence |
| Retz 2021 | Attention-deficit/hyperactivity disorder (ADHD), antisociality and delinquent behavior over the lifespan | Neuroscience and Biobehavioral Reviews | 120 | 236-248 | Not empirical |
| Retz-Junginger 2014 | ADHD and child sexual abuse | Zeitschrift Fur Psychiatrie Psychologie Und Psychotherapie |  | 175-181 | Not on violence |
| Rey 2005 | Similarities and differences between aggressive and delinquent children and adolescents in a national sample | The Australian and New Zealand Journal of Psychiatry | 39 | 366-372 | Not on violence |
| Robert 2015 | Forensische kinder-, jeugd- en transitiepsychiatrie |  |  |  | Not empirical |
| Rodrigues Stefanini 2015 | Adolescents with attention deficit hyperactivity disorder and exposure to violence: parents' opinion | Revista Latino-Americana De Enfermagem | 23 | 1090-1096 | Not empirical |
| Roe-Sepowitz 2008 | Examining the sexual offenses of female juveniles: the relevance of childhood maltreatment | The American Journal of Orthopsychiatry | 78 | 405-412 | Not on ADHD |
| Roesler 2008 | On the relations between attention deficit-/hyperactivity disorder, antisocial personality disorder, and delinquency | Zeitschrift Fur Psychiatrie Psychologie Und Psychotherapie | 56 | 121-130 | Not empirical |
| Rokeach | The romantic relationships of adolescents with attention-deficit/hyperactivity disorder (ADHD) |  |  |  | Inadequate comparator |
| Rokeach 2018 | The romantic relationships of adolescents with ADHD | Journal of Attention Disorders ; Volume 22, Issue 1, Page 35-45 ; ISSN 1087-0547 1557-1246 | 22 | 35-45 | No association data |
| Rolland 2015 | Attention-deficit/hyperactivity disorder (ADHD) in adults: specific clinical and therapeutic issues | European Psychiatry | 30 | S27-S28 | Not empirical |
| Román-Ithier 2017 | Attention deficit hyperactivity disorder symptoms, type of offending and recidivism in a prison population: the role of substance dependence | Criminal Behaviour and Mental Health | 27 | 443-456 | Not on violence |
| Romero-Martinez 2016 | Testosterone and attention deficits as possible mechanisms underlying impaired emotion recognition in intimate partner violence perpetrators | European Journal of Psychology Applied to Legal Context | 8 | 57-62 | Not on ADHD |
| Romero-Martinez 2019 | The importance of impulsivity and attention switching deficits in perpetrators convicted for intimate partner violence | Aggressive Behavior | 45 | 129-138 | Not on ADHD |
| Romero-Martinez 2020 | Alexithymia as a predictor of arousal and affect dysregulations when batterers with attention deficit hyperactivity disorder cope with acute stress | Behavioral Sciences | 10 |  | Inadequate comparator |
| Sacchetti 2014 | ADHD symptomology and social functioning in college students | Journal of Attention Disorders ; Volume 21, Issue 12, Page 1009-1019 ; ISSN 1087-0547 1557-1246 | 21 | 1009-1019 | Not on ADHD |
| Sacchetti 2014 | Anger, relationships, and intimate partner violence in emerging adults with and without ADHD symptomology | University of Northern Iowa, Rod Library |  |  | Not on ADHD |
| Schlack 2013 | Psychological problems, protective factors and health-related quality of life in youth affected by violence: the burden of the multiply victimised | Journal of Adolescence | 36 | 587-601 | Not on violence |
| Schmidt 2014 | Integrating families into treatment for adolescents with illegal sexual behavior |  |  |  | Not empirical |
| Schubert 2011 | Influence of mental health and substance use problems and criminogenic risk on outcomes in serious juvenile offenders | Journal of the American Academy of Child and Adolescent Psychiatry | 50 | 925-937 | Not on violence |
| Schubert 2011 | Influence of mental health and substance use problems and criminogenic risk on outcomes in serious juvenile offenders | Journal of the American Academy of Child & Adolescent Psychiatry | 50 | 925-937 | Not on violence |
| Smith 2007 | ADHD and risky sexual behavior |  |  |  | Not on violence |
| Snyder 2019 | Examining the link between assault victimization and ADHD among college men | Criminal Justice Studies | 32 | 16-31 | Not on violence |
| Steele 2020 | Birds of a feather: an examination of ADHD symptoms and associated concerns in partners of adults with ADHD | Journal of Attention Disorders |  | 1087054720978553-1087054720978553 | Not on ADHD |
| Sultan 2021 | Adolescents with attention-deficit/hyperactivity disorder: adverse behaviors and comorbidity | The Journal of Adolescent Health | 68 | 284-291 | Not on violence |
| Taylor 1996 | Hyperactivity and conduct problems as risk factors for adolescent development | Journal of the American Academy of Child and Adolescent Psychiatry | 35 | 1213-1226 | Not on violence |
| Taylor 2010 | In harm’s way: adults with attention-deficit/ hyperactivity disorder in high-risk situations | European Child & Adolescent Psychiatry | 19 | S67-S67 | Not on violence |
| Theriault 2001 | Impulsive, but violent? Are components of the attention deficit-hyperactivity syndrome associated with aggression in relationships? | Violence Against Women | 7 | 1464-1489 | Not on ADHD |
| Tiegs 2014 | New problems arising from old drugs: second-generation effects of acetaminophen | Expert Review of Clinical Pharmacology | 7 | 655-662 | Not empirical |
| Troy 2021 | Childhood psychopathology mediates associations between childhood adversities and multiple health risk behaviours in adolescence: analysis using the ALSPAC birth cohort | Journal of Child Psychology and Psychiatry |  | 1100-1109 | Not on violence |
| University 2010 | Committed romantic relationships in couples with ADHD: subtypes, conflict resolution and satisfaction |  |  |  | Not on violence |
| Vaih-Koch 2001 | ADHD und störung des sozialverhaltens im kindesalter als prädiktoren aggressiver sexualdelinquenz? = ADHD and conduct disorder in childhood as predictors of aggressive sexual delinquency? | Sexuologie: Zeitschrift Für Sexualmedizin, Sexualtherapie Und Sexualwissenschaft | 8 | ene-18 | Inadequate comparator |
| van Vugt 2021 | Similarities and differences between youth who engaged in intrafamilial and extrafamilial sexually abusive behavior: an exploratory study | International Journal of Offender Therapy and Comparative Criminology | 65 | 51-67 | Inadequate comparator |
| Van Wijk 2007 | Incarcerated Dutch Juvenile Sex Offenders Compared with Non-Sex Offenders | Journal of Child Sexual Abuse | 16 | 1-21 | Inadequate comparator |
| VanderDrift 2019 | Inattention and hyperactivity-impulsivity: their detrimental effect on romantic relationship maintenance | Journal of Attention Disorders | 23 | 985-994 | Not on violence |
| Vrijsen 2018 | ADHD symptoms in healthy adults are associated with stressful life events and negative memory bias | ADHD Attention Deficit and Hyperactivity Disorders |  | 151-160 | Not on violence |
| Wallin 2022 | Self-experienced sexual and reproductive health in young women with attention deficit hyperactivity disorder a qualitative interview study | Bmc Womens Health |  |  | Not empirical |
| Walters 2021 | Relationships, sexuality, and sexual behavior in adolescents with ADHD - chapter 17 |  |  |  | Not empirical |
| White 2012 | Adolescent sexual victimization, ADHD symptoms, and risky sexual behavior | Journal of Family Violence | 27 | 123-132 | Inadequate comparator |
| White 2014 | Childhood attention deficit hyperactive disorder (ADHD) symptoms and adolescent female sexual victimisation: mediating and moderating effects of risky behaviours | Journal of Sexual Aggression |  | 23-39 | Not on ADHD |
| Williams 2019 | Medication moderates link between ADHD and some intimate partner violence measures | Posters |  |  | Not on ADHD |
| Witt 2016 | Data on maltreatment profiles and psychopathology in children and adolescents | Data In Brief | 8 | 1352-1356 | Not on violence |
| Wojciechowski 2021 | The role of ADHD in predicting the development of violent behavior among juvenile offenders: participation versus frequency | Journal of Interpersonal Violence | 36 | NP625-NP642 | Not on violence |
| Wolf 2008 | Differentiating two types of juvenile sex offenders |  |  |  | Inadequate comparator |
| Wong 2022 | Associations between childhood maltreatment and psychiatric disorders: analysis from electronic health records in Hong Kong | Translational Psychiatry |  |  | Not on violence |
| Wood 2021 | Health behaviors of college students with mental health conditions | International Journal of Health, Wellness & Society | 11 | 59-68 | Not on ADHD |
| Wu 2021 | Association between hyperactivity symptoms and somatic complaints: mediating and moderating mechanisms in childhood trauma and life events among Chinese male adolescents | Frontiers In Psychiatry | 12 | 44470 | Not on violence |
| Wymbs 2009 | Link between problem drinking and intimate partner violence is stronger among young adults with childhood attention-deficit hyperactivity disorder | Alcoholism-Clinical and Experimental Research | 33 | 251A-251A | No association data |
| Wymbs 2014 | Do elevated ADHD symptoms increase the risk of young adult men perpetrating partner violence? ; (539122014-001) |  |  |  | Not on ADHD |
| Wymbs 2014 | Childhood ADHD potentiates the association between problematic drinking and intimate partner violence | Journal of Attention Disorders | 21 | 997-1008 | No association data |
| Wymbs 2015 | Integrative couples group treatment for emerging adults with ADHD symptoms | Cognitive and Behavioral Practice | 22 | 161-161 | Not on violence |
| Wymbs 2017 | ADHD symptoms as risk factors for intimate partner violence perpetration and victimization | Journal of Interpersonal Violence | 32 | 659-681 | No association data |
| Wymbs 2019 | Rates of intimate partner violence perpetration and victimization among adults with ADHD | Journal of Attention Disorders | 23 | 949-958 | No association data |
| Wymbs 2021 | Testing ADHD and additional moderators of the causal association between acute alcohol intoxication and lab-based intimate partner aggression | Alcoholism-Clinical and Experimental Research | 45 | 242A-242A | Not on ADHD |
| Wymbs 2021 | Adult ADHD and romantic relationships: what we know and what we can do to help | Journal of Marital and Family Therapy | 47 | 664-681 | Not empirical |
| Yeh 2017 | Pain, bullying involvement, and mental health problems among children and adolescents with ADHD in Taiwan | Journal of Attention Disorders | 23 | 809-816 | Not on violence |
| Yoo 2021 | Association between attention deficit hyperactivity disorder and aggression subscales in adolescents | Brain and Behavior | 11 | e02030-e02030 | Not on violence |
| Yoshihiro 2015 | The Relationship Between Bullying and Victimization, and Traits of Autism Spectrum Disorder and Attention Deficit/Hyperactivity Disorder In School Children. |  | 26 | 332-343 | Not on violence |
| Youn 2018 | Childhood social functioning and young adult intimate partner violence in girls with and without ADHD: response inhibition as a moderator | Journal of Attention Disorders | 23 | 1486-1496 | Not on ADHD |
| Young 2005 | The adolescent outcome of hyperactive girls: self-report of psychosocial status | Journal of Child Psychology and Psychiatry, and Allied Disciplines | 46 | 255-262 | Not on violence |
| Zalecki 2004 | Overt and relational aggression in girls with attention deficit hyperactivity disorder | Journal of Clinical Child and Adolescent Psychology | 33 | 125-137 | Not on violence |
| Zendarski 2021 | Peer victimization and poor academic outcomes in adolescents with ADHD: what individual factors predict risk? | Journal of Attention Disorders | 25 | 1455-1465 | Not on violence |

**Table S8 Studies reported over multiple publications**

| **Study** | **Notes** | **Publications** |
| --- | --- | --- |
| **Snyder 2015** | **The study was published both as a doctoral dissertation and as a scientific article** | Snyder, J. A. (2015). The Link Between ADHD and the Risk of Sexual Victimization Among College Women: Expanding the Lifestyles/Routine Activities Framework. Violence Against Women, 21(11), 1364–1384. <https://doi.org/10.1177/1077801215593647>  Snyder, J. A. (2011). College Students with ADHD: Extending the Lifestyles/Routine Activities Framework to Predict Sexual Victimization and Physical Assault. University of Cincinnati. |
| **Guendelman 2016** | **The study was published both as a doctoral dissertation and as a scientific article** | Guendelman, M. D., Ahmad, S., Meza, J. I., Owens, E. B., & Hinshaw, S. P. (2016). Childhood Attention-Deficit/Hyperactivity Disorder Predicts Intimate Partner Victimization in Young Women. Journal of Abnormal Child Psychology, 44(1), 155–166. <https://doi.org/10.1007/s10802-015-9984-z>  Stier, A. L. (2009). UC Berkeley UC Berkeley Electronic Theses and Dissertations [University of California, Berkeley]. In The Influence of ADHD and Adolescent Romantic Relationships on Early Adult Psychopathology in Females. https://escholarship.org/uc/item/98384265 |
| **Buitelaar 2020** | **The study was published both as a doctoral dissertation and as a scientific article.**  **While this study was not formally included in our meta-analysis, it is a key previous systematic review** | Buitelaar, N. J. L., Posthumus, J. A., & Buitelaar, J. K. (2020). ADHD in Childhood and/or Adulthood as a Risk Factor for Domestic Violence or Intimate Partner Violence: A Systematic Review. Journal of Attention Disorders, 24(9), 1203–1214. <https://doi.org/10.1177/1087054715587099>  Buitelaar, N. J. L. (2020). ADHD and intimate Partner Violence. Impact of ADHD as a risk and a treatment factor in Intimate Partner Violence. Radboud Universit. |

**Table S9 Details on the databases used in the studies**

| **Author year** | **Database acronym** | **Database** |
| --- | --- | --- |
| Blocher 2001 |  | Longitudinal study on ADHD in inmates |
| Campe 2021 | ACHA-NCHA-2016 | American College Health Association, National College Health Assessment |
| Crane 2014 |  | Connecticut presentencing trial phase evaluations for substance abuse |
| Fang 2010 | Add Health | National Longitudinal Study of Adolescent Health |
| González 2013 | APMS, 2007 | Adult Psychiatric Morbidity Survey (2007) |
| Guendelman 2016 | BGALS | Berkeley Girls with ADHD Longitudinal Study |
| McCauley 2015 | NCS-R | National Comorbidity Survey Replication |
| Mohr-Jensen 2019 | DPCRR and DNPR | Danish National Registries: Danish Psychiatric Central Research Register (DPCRR) or the Danish National Patient Register (DNPR). |
| Ngo 2018 | SSLS | Secondary Student Life Survey |
| Scherer 2016 | ACHA-NCHA II-2008 | American College Health Association’s National College Health Assessment II |
| Snyder 2015 | ACHA-NCHA II-2008 | American College Health Association’s National College Health Assessment II; 2008 |
| Wymbs 2012 | PALS; STP | Pittsburgh ADHD Longitudinal Study; Summer Treatment Programm |
| Wymbs 2021 | MTurk | Amazon’s Mechanical Turk |
| Yu 2019 | Swedish national registries | National Patient Register, National Crime Register, the Multi-Generation Register (Statistics Sweden), Longitudinal Integration Database for Health Insurance and Labour Market Studies |

**Table S10 Details on setting, identification of ADHD and identification of violence in the studies**

| **Author year** | **Setting** | **Identification of ADHD** | | | | **Identification of violence** | |
| --- | --- | --- | --- | --- | --- | --- | --- |
|  |  | **Diagnostic manual** | **Tool** | **When diagnosis respect to violence** | **When diagnosis (age/year range)** | **Tool** | **When identification (age/year range)** |
| Blocher 2001 | Other (inmates) | ICD-10 | WURS | Not concurrent to IPV | Symptoms in childhood evaluated in adulthood |  | NR |
| Campe 2021 | Epidemiological | NA | NA: Self-reported | Not concurrent to IPV | NA |  | The past 12 months |
| Crane 2014 | Other (criminal offenders with suspected substance use) | NR | NA: Clinical diagnosis | Not concurrent to IPV | Past 5 years |  | Past year |
| Fang 2010 | Epidemiological | DSM-IV | Unnamed symptom scale | Not concurrent to IPV | Symptoms in childhood evaluated in adulthood |  | Past 6 years |
| González 2013 | Epidemiological | DSM-IV | ASRS-6 | Concurrent to IPV | Past 6 months |  | Past 5 years |
| Guendelman 2016 | Other (participants in ADHD summer camp) | DSM-IV | DISC-IV, SNAP-IV | Not concurrent to IPV | Wave 1 y Wave 3 (at age 10) | Health and Sexual Behavior Questionnaire (HSBQ) | Wave 3 (10 years from wave 1) |
| McCauley 2015 | Epidemiological | DSM-IV and ICD-10 | WMH-CIDI | Not concurrent to IPV | Prior to dating initiation | Conflict Tactics Scale (CTS) | Prior to age 21 |
| Mohr-Jensen 2019 | Epidemiological | ICD-10 | NA: Administrative datatabase | Not concurrent to IPV | 1995-2005; between 4y and 15y |  | Until December 2014 |
| Ngo 2018 | Epidemiological | DSM-IV | YSR/11-18 | Concurrent to IPV | NA |  | Last year |
| Scherer 2016 | Epidemiological | NA | NA: Self-reported | Concurrent to IPV | NA |  | Last year |
| Snyder 2015 | Epidemiological | NA | NA: Self-reported | Concurrent to IPV | 1089: ADHD diagnosed anywhen; 780 within last 12 months; 582 within last 12 months and currently under treatment for ADHD. |  | Last year |
| Wymbs 2012 | Other (participants in ADHD summer camp) | DSM-III-R, DSM-IV | NA: Clinical diagnosis | Not concurrent to IPV | 1987 -1996. Mean age 9.40 (SD = 2.27) with 90% between the ages of 5 and 12 | Conflict Tactics Scale (CTS) | Last year |
| Wymbs 2021 | Epidemiological | DSM-IV | CAARS-LF | Not concurrent to IPV |  | Sexual Experiences Survey– Short Form Victimization (SES-SFV) | At or after 14 years |
| Yu 2019 | Epidemiological | ICD-10 | NA: Administrative datatabase | Not concurrent to IPV | Before arrest for IPV during 1998–2013 | Crime Code (arrests) from national registry | Between 1 January 1998 and 31 December 2013. Individuals aged 15 years (the age of criminal responsibility) and older. |

**Table S11 Details on the overall samples of the studies**

| **Author year** | **N** | **Age** | **Sex (% male)** | **Socioeconomic status** | **Ethnicity** | **Comorbidities** | **Medication status** | **Variables controlled on design** |
| --- | --- | --- | --- | --- | --- | --- | --- | --- |
| Blocher 2001 | 294 | NR | 100 | NR | NR | NR | NR | NR |
| Campe 2021 | 22,828 | NR | 0 | NR | 68.9% White/Caucasian |  | NR | Substance use, sociodemographic variables |
| Crane 2014 | 190 | M=30.44 (SD=10); 18-59 | 66 | 35% employed | Caucasian (78%), African American (12%) Hispanic (7%), and other (3%) ethnicities. | 100% substance use disorders | NR | Gender, ethnicity and substance use diagnoses |
| Fang 2010 | 11,238 | M=22 | 49.6 | Primary caregiver education, % (95% CI), <High school 15.2 (14.5-15.9), High school or equivalent 33.0 (32.2-33.9), Some college 27.9 (27.1-28.8), >College graduate 23.8 (23.0-24.6); Family income index, % (95% CI), <1 FPL 13.6 (13.0-14.2), 1 to <2 FPL 18.2 (17.5-18.9), 2 to <4 FPL 44.8 (43.9-45.8), >4 FPL 23.4 (22.6-24.2) | Hispanic 11.5 (10.9-12.1), Non-Hispanic white 68.8 (68.0-69.7), Non-Hispanic black 14.6 (14.0-15.3), Non-Hispanic other 5.1 (4.7-5.4) | ADHD diagnostic category, % (95% CI): 8.4 (7.8-8.9); CD diagnostic category, % (95% CI): 12.4 (11.8-13.0) | NR | None |
| González 2013 | 7,369 | NR | NR | NR | NR | NR | NR | NR |
| Guendelman 2016 | 193 | M=9.5 (SD=1.7) at diagnosis, with 10 year follow up | 0 | Mean=6.6. SD=2.6; in a scale in which for total annual family income, 1 ≤ $10,000; 9 ≥ $75,000 | 55.4 Caucasian | Low birth weight (<2500 g), WISC-III full scale IQ, Reading disorderd , Math disordere, Diagnosis ofOppositional Defiant Disorder and/or Conduct Disorder, Anxiety Disorder and/or Major Depressive Episode and/or Dysthymia ADHD-related | NR | NR |
| McCauley 2015 | 5,112 | >21 (dating before age 21) | NR | NR | NR | NR | NR | NR |
| Mohr-Jensen 2019 | 23,826 | NR | NR | NR | NR | NR | NR | Sex and age matched |
| Ngo 2018 | 4,665 | 12-18 | 48.9 | Both parents have less than a college degree 24.5%, At least one parent has a college degree or higher 75.5%; High to moderate income school district 57.9%, Low income school district 42.1% | White 64.4%, Black 30.3%, ‘Other race’ 5.3% | Potential substance use disorders: Positive DAST-10 screen (2+ items) 12.9%, Positive CRAFTT screen (2+ items) 17.7%; Depression 9.5%, Anxiety 4.3%, ADHD 4.8%, Conduct disorder 5.5% | NR | None |
| Scherer 2016 | 20,486 | M=19.63 (SD=1.56); 18-25 | 30.89 | NR | White 70.90%; Non-White 29.10% | Mental disabilities (n = 967) and physical disabilities (n = 583) | NR | None |
| Snyder 2015 | 14,816 | M=19.6; 18-24 | 0 | NR | 77% white | NR | NR | NR |
| Wymbs 2012 | 231 | 18-25 | 100 | NR | NR | NR | NR | NR |
| Wymbs 2021 | 218 | 18-45 | 55% | NR | NR | NR | NR | NR |
| Yu 2019 | 1,025,450 | NR | NR | NR | NR | NR | NR | Age and sex |

**Table S12 Data on the ADHD samples**

| **Author year** | **N** | **Mean age** | **SD age** | **Other age data** | **Sex (% male)** | **Socioeconomic status** | **Ethnicity** | **Comorbidities** | **Medication status** |
| --- | --- | --- | --- | --- | --- | --- | --- | --- | --- |
| Blocher 2001 | NR | NR | NR | NR | NR | NR | NR | NR | NR |
| Campe 2021 | 1,566 | NR | NR | NR | 0 | NR | NR | Chronic illness 6.18%, deaf/hard hearing 1.62%, learning disability 4.21%, mobility disability 0.85%, blind 2.36%, psychiatric condition 8.96%, language disorder 0.66, other 2.19% | NA |
| Crane 2014 | 32 | NR | NR | NR | 91% | 27% employed | Caucasian (78%), African American (1%) Hispanic (16%), and other (5%) ethnicities. | 100% substance use disorders (alcohol 62%, cocaine 56%, marijuana 72%, opiate 56%) | NR |
| Fang 2010 | 944 | NR | NR | NR | NR | NR | NR | NR | NR |
| González 2013 | 424 | NR | NR | 16-14: 189 (8,3%); 35-54: 171 (6.5%); >54: 64 (2.6%) | 52% | I&II n=110; III (M&NM) n=158; IV&V n=101: Social class was based on the UK Registrar General’s Classification [36] which uses the most recent occupation of the head of household: I - professional, II - managerial, IIIA - skilled non-manual, IIIB - skilled manual, IV – partly skilled, V- unskilled. These were combined in three categories: I & II (upper middle class), III (lower middle and skilled working class) and IV & V (less skilled and unskilled) | White n=366, black/African origin n=22, Indian subcontinent n=10, other n=20 | Antisocial personality disorder n=45, drug dependence n=46, alcohol dependence n=74, anxiety disorders n=228, psychosis n=33 | NR |
| Guendelman 2016 | 114 | 9.7 | 1.7 | Age at diagnosis (wave 1) | 0 | Mean=6.4. SD=2.7; in a scale in which for total annual family income, 1 ≤ $10,000; 9 ≥ $75,000 | 59.6% Caucasian | Oppositional Defiant Disorder and/or Conduct Disorder, Anxiety Disorder and/or Major Depressive Episode and/or Dysthymia | NR |
| McCauley 2015 | 311 | NR | NR | NR | 49.5% | NR | NR | NR | NR |
| Mohr-Jensen 2019 | 4,231 | 22 | 5.8 | NR | 85.2% | Mean family income first 10 y Lowest third 2,189, Middle third 1,119, Highest third 815 | NR | Substance use disorder 7.3%, Psychotic disorder Depression 2.9%, Depression 3.6%, Anxiety disorder 12.2%, Personality disorder Intellectual disability 3.3%, Intellectual disability 15.4%, Specific disorder of development 31.6%, Autism Spectrum disorder 19.9%, Conduct disorder/ Oppositional Defiant Disorder 34.7%, Tic disorder/Tourette’s disorder 7.8% | Any ADHD medication 70.9%, SSRIs 19.7% |
| Ngo 2018 | NR | NR | NR | NR | NR | NR | NR | NR | NR |
| Scherer 2016 | 1,459 | NR | NR | NR | NR | NR | NR | NR | NR |
| Snyder 2015 | 652 | NR | NR | NR | NR | NR | NR | NR | NR |
| Wymbs 2012 | 125 | 19.98 | 1.96 | 18-25 | 100 | NR | NR | NR | NR |
| Wymbs 2021 | 97 | 28.03 | 5.03 | 18-45 | 55.67 | Highest level of education: 44.33% associate’s degree or higher | 84.54% Caucasian | NR | NR |
| Yu 2019 | 49,327 | NR | NR | mean age at start of follow-up: 23.3 (SD=11.5) | NR | Low income 45.5% | Born abroad 8% | NR | NR |

**Table S13 Data on the non-ADHD sample (ADHD comparators)**

| **Author year** | **N** | **Mean age** | **SD age** | **Other age data** | **Sex (% male)** | **Socioeconomic status** | **Ethnicity** | **Comorbidities** | **Medication status** |
| --- | --- | --- | --- | --- | --- | --- | --- | --- | --- |
| Blocher 2001 | NR | NR | NR | NR | NR | NR | NR | NR | NR |
| Campe 2021 | 21,262 | NR | NR | NR | NR | College | NR | NR | NR |
| Crane 2014 | 32 | NR | NR | NR | 88% | 50% employed | Caucasian (78%), African American (6%) Hispanic (16%), and other (5%) ethnicities. | 100% Substance use disorders (alcohol 63%, cocaine 56%, marijuana 69%, opiate 56%) | NR |
| Fang 2010 | 10294 | NR | NR | NR | NR | NR | NR | NR | NR |
| González 2013 | NR | NR | NR | NR | NR | NR | NR | NR | NR |
| Guendelman 2016 | 79 | 9.4 | 1.7 | Age at diagnosis (wave 1) | 0 | Mean=6.9. SD=2.4; in a scale in which for total annual family income, 1 ≤ $10,000; 9 ≥ $75,000 | 49.4 Caucasian | Oppositional Defiant Disorder and/or Conduct Disorder, Anxiety Disorder and/or Major Depressive Episode and/or Dysthymia | NR |
| McCauley 2015 | NR | NR | NR | NR | NR | NR | NR | NR | NR |
| Mohr-Jensen 2019 | 19,595 | 22.1 | 5.8 | NR | 84.8% | Mean family income first 10 y Lowest third 6,409, Middle third 6,925, Highest third 6,169 | NR | Substance use disorder 2.1%, Psychotic disorder Depression 0.7%, Depression 1%, Anxiety disorder 3%, Personality disorder Intellectual disability 0.5%, Intellectual disability 0.9%, Specific disorder of development 0.7%, Autism spectrum disorder 1%, Conduct disorder/ oppositional defiant disorder 0.9%, Tic disorder/Tourette’s disorder 0.5% | SSRIs 6.9% |
| Ngo 2018 | NR | NR | NR | NR | NR | NR | NR | NR | NR |
| Scherer 2016 | NR | NR | NR | NR | NR | NR | NR | NR | NR |
| Snyder 2015 | 14,019 | NR | NR | NR | NR | NR | NR | NR | NR |
| Wymbs 2012 | 88 | 19.76 | 1.72 | 18-25 | 100 | NR | NR | NR | NR |
| Wymbs 2021 | 121 | 28.12 | 5.75 | 18-45 | 54.55 | Highest level of education: 52.89% associate’s degree or higher | 80.99% Caucasian | NR | NR |
| Yu 2019 | 976,123 | NR | NR | Mean age at start of follow-up: 23.3 (SD=11.4) | NR | Low income 26.9% | Born abroad 7.3% | NR | NR |

**Table S14 Risk of bias evaluation**

**Number of stars for each item of the Newcastle-Ottawa Scale divided according to the type of study**

|  | **SELECTION** | | | | **COMPARABILITY** | **EXPOSURE** | | | **TOTAL** |
| --- | --- | --- | --- | --- | --- | --- | --- | --- | --- |
| **Item** | **1** | **2** | **3** | **4** | **1** | **1** | **2** | **3** |  |
| **Case-control studies** |  |  |  |  |  |  |  |  |  |
| Blocher 2001 | 1 | 0 | 0 | 1 | 0 | 0 | 0 | 0 | 2 |
|  |  |  |  |  |  |  |  |  |  |
| **Cohort studies** |  |  |  |  |  |  |  |  |  |
| Guendelman 2016 | 0 | 0 | 1 | 0 | 2 | 0 | 1 | 0 | 4 |
| Mohr-Jensen 2019 | 1 | 1 | 1 | 1 | 2 | 1 | 1 | 1 | 9 |
| Yu 2019 | 1 | 1 | 1 | 1 | 2 | 1 | 1 | 1 | 9 |
|  |  |  |  |  |  |  |  |  |  |
| **Cross-sectional studies** |  |  |  |  |  |  |  |  |  |
| Campe 2021 | 0 | 1 | 0 | 0 | 2 | 0 | 1 | NA | 4 |
| Crane 2014 | 0 | 0 | 0 | 1 | 1 | 0 | 1 | NA | 3 |
| Fang 2010 | 1 | 1 | 1 | 0 | 1 | 0 | 1 | NA | 5 |
| González 2013 | 1 | 1 | 1 | 0 | 2 | 0 | 1 | NA | 6 |
| McCauley 2015 | 1 | 1 | 0 | 2 | 2 | 1 | 1 | NA | 8 |
| Ngo 2018 | 1 | 1 | 1 | 0 | 2 | 0 | 1 | NA | 6 |
| Scherer 2016 | 1 | 1 | 0 | 0 | 2 | 0 | 1 | NA | 5 |
| Snyder 2015 | 1 | 1 | 0 | 0 | 1 | 0 | 1 | NA | 4 |
| Wymbs 2012 | 0 | 0 | 0 | 1 | 1 | 1 | 0 | NA | 3 |
| Wymbs 2021 | 0 | 0 | 0 | 0 | 1 | 1 | 1 | NA | 3 |

**Table S15 Effect size data**

| **Author year** | **Type of Violence** | **Subtype of violence** | **Type of involvement** | **Additional description** | **Effect size ID** | **N with ADHD&IPV/SV** | **N with ADHD (No IPV/SV)** | **N with IPV/SV (no ADHD)** | **N without ADHD or IPV/SV** | **N ADHD** | **N IPV/SV** | **TOTAL N** |
| --- | --- | --- | --- | --- | --- | --- | --- | --- | --- | --- | --- | --- |
| Blocher 2001 | SV | SV in general | As perpetrator |  | 1 | 35 | 13 | 92 | 154 | 48 | 127 | 294 |
| Campe 2021 | SV | SV in general | As victim | Completed sexual assault | 2 | NR | NR | NR | NR | 1,566 | 685 | 22,828 |
| Campe 2021 | IPV | Rape/penetration SV | As victim |  | 3 | NR | NR | NR | NR | 1,566 | 637 | 22,828 |
| Crane 2014 | IPV | Physical | As perpetrator |  | 4 | 8 | 24 | 5 | 27 | 32 | 13 | 64 |
| Fang 2010 | IPV | Physical | As perpetrator | IPV without and with injuries combined (unadjusted) | 5 | 303 | 641 | 2,742 | 7,552 | 944 | 3,045 | 11,238 |
| Fang 2010 | IPV | Physical | As perpetrator | IPV without injury vs never (adjusted) | 6 | 223 | 641 | 2,204 | 7,552 | 864 | 2,427 | 10,620 |
| González 2013 | IPV | Physical | As perpetrator |  | 7 | NR | NR | NR | NR | 424 | 115 | 7,369 |
| Guendelman 2016 | IPV | Physical | As victim |  | 8 | 35 | 79 | 5 | 74 | 114 | 40 | 193 |
| Guendelman 2016 | IPV | Physical | As perpetrator |  | 9 | 13 | 79 | 3 | 57 | 92 | 16 | 152 |
| McCauley 2015 | IPV | Physical | As perpetrator |  | 10 | NR | NR | NR | NR | NR | NR | NR |
| McCauley 2015 | IPV | Physical | As victim |  | 11 | NR | NR | NR | NR | NR | NR | NR |
| Mohr-Jensen 2019 | SV | SV in general | As perpetrator |  | 12 | 109 | 4,122 | 95 | 19,500 | 4,231 | 204 | 23,826 |
| Ngo 2018 | SV | SV in general | As victim | Females | 13 | NR | NR | NR | NR | NR | 1,275 | 2,383 |
| Ngo 2018 | SV | SV in general | As perpetrator | Females | 14 | NR | NR | NR | NR | NR | 300 | 2,383 |
| Ngo 2018 | SV | SV in general | As victim | Males | 15 | NR | NR | NR | NR | NR | 774 | 2,282 |
| Ngo 2018 | SV | SV in general | As perpetrator | Males | 16 | NR | NR | NR | NR | NR | 520 | 2,282 |
| Scherer 2016 | IPV | IPV in general | As victim |  | 17 | 246 | 1,213 | 1,688 | 17,339 | 1,459 | 1,934 | 20,486 |
| Scherer 2016 | IPV | Physical | As victim |  | 18 | 67 | 1,390 | 338 | 18,691 | 1,457 | 405 | 20,486 |
| Scherer 2016 | IPV | SV in general | As victim |  | 19 | 39 | 1,411 | 198 | 18,838 | 1,450 | 237 | 20,486 |
| Snyder 2015 | SV | SV in general | As victim |  | 20 | 108 | 547 | 1,444 | 12,575 | 655 | 1,552 | 14,674 |
| Wymbs 2012 | IPV | Psychological | As perpetrator |  | 21 | 29 | 96 | 7 | 81 | 125 | 36 | 213 |
| Wymbs 2021 | SV | SV in general | As victim |  | 22 | 52 | 45 | 36 | 85 | 97 | 88 | 218 |
| Yu 2019 | IPV | IPV in general | As perpetrator |  | 23 | 296 | 49,031 | 976 | 975,147 | 49,327 | 1,272 | 1,025,450 |

**Table S16 Effect sizes; analyses index**

| **Author year** | **Effect size ID** | **Effect size** | **IPV perpetrator** | **SV perpetrator** | **IPV victim** | **SV victim** | **SV victim; females** | **IPV physical perpetrator** | **IPV physical victim** | **IPV perpetrator; cross sectional** | **SV victim; cross sectional** | **IPV perpetrator; population-based** | **SV victim; population-based** | **IPV perpetrator; quality diagnosis** | **IPV perpetrator; adjusted** | **IPV victim; adjusted** | **SV perpetrator; adjusted** | **SV victim; adjusted** |
| --- | --- | --- | --- | --- | --- | --- | --- | --- | --- | --- | --- | --- | --- | --- | --- | --- | --- | --- |
| Blocher 2001 | 1 | OR=4.51(2.27-8.96) |  | X |  |  |  |  |  |  |  |  |  |  |  |  |  |  |
| Campe 2021 | 2 | aOR=1.43(1.01-2.03) |  |  |  | X | X |  |  |  | X | X | X |  |  |  |  | X |
| Campe 2021 | 3 | aOR=1.25(0.87-1.77) |  |  | X |  |  |  |  |  |  |  |  |  |  | X |  |  |
| Crane 2014 | 4 | OR=1.88(0.54-6.54) | X |  |  |  |  | X |  | X |  |  |  | X |  |  |  |  |
| Fang 2010 | 5 | OR=1.3(1.13-1.5) | X |  |  |  |  | X |  | X |  | X |  |  |  |  |  |  |
| Fang 2010 | 6 | aOR=1.09(0.87-1.35) |  |  |  |  |  |  |  |  |  |  |  |  | X |  |  |  |
| González 2013 | 7 | aOR=1.52(0.7-3.28) | X |  |  |  |  | X |  | X |  | X |  |  | X |  |  |  |
| Guendelman 2016 | 8 | OR=6.56(2.44-17.63) |  |  | X |  |  |  | X |  |  |  |  |  |  |  |  |  |
| Guendelman 2016 | 9 | OR=3.13(0.85-11.48) | X |  |  |  |  | X |  |  |  |  |  |  | X |  |  |  |
| McCauley 2015 | 10 | aOR=2.22(1.7-2.9) | X |  |  |  |  | X |  | X |  | X |  | X | X |  |  |  |
| McCauley 2015 | 11 | aOR=1.65(1.19-2.28) |  |  | X |  |  |  | X |  |  |  |  |  |  | X |  |  |
| Mohr-Jensen 2019 | 12 | OR=5.43(4.12-7.16) |  | X |  |  |  |  |  |  |  |  |  |  |  |  |  |  |
| Ngo 2018 | 13 | aOR=1.72(1.17-2.54) |  |  |  | X | X |  |  |  |  |  |  |  |  |  |  | X |
| Ngo 2018 | 14 | aOR=1.72(1.02-2.9) |  | X |  |  |  |  |  |  |  |  |  |  |  |  | X |  |
| Ngo 2018 | 15 | aOR=1.54(1.04-2.29) |  |  |  | X |  |  |  |  | X |  | X |  |  |  |  | X |
| Ngo 2018 | 16 | aOR=1.31(0.845-2.05) |  | X |  |  |  |  |  |  |  |  |  |  |  |  | X |  |
| Scherer 2016 | 17 | aOR=1.39(1.16-1.65) |  |  | X |  |  |  |  |  |  |  |  |  |  | X |  |  |
| Scherer 2016 | 18 | OR=2.67(2.04-3.49) |  |  |  |  |  |  | X |  |  |  |  |  |  |  |  |  |
| Scherer 2016 | 19 | OR=2.63(1.86-3.72) |  |  |  | X |  |  |  |  |  |  |  |  |  |  |  |  |
| Snyder 2015 | 20 | OR=1.72(1.39-2.13) |  |  |  | X | X |  |  |  | X |  | X |  |  |  |  |  |
| Wymbs 2012 | 21 | OR=3.5(1.46-8.41) | X |  |  |  |  |  |  | X |  |  |  | X |  |  |  |  |
| Wymbs 2021 | 22 | OR=2.73(1.56-4.77) |  |  |  | X |  |  |  |  | X |  | X |  |  |  |  |  |
| Yu 2019 | 23 | aHR=6.4(5.5-7.4) | X |  |  |  |  |  |  |  |  |  |  | X | X |  |  |  |

**Figure S1 IPV perpetrator; funnel plot**

**
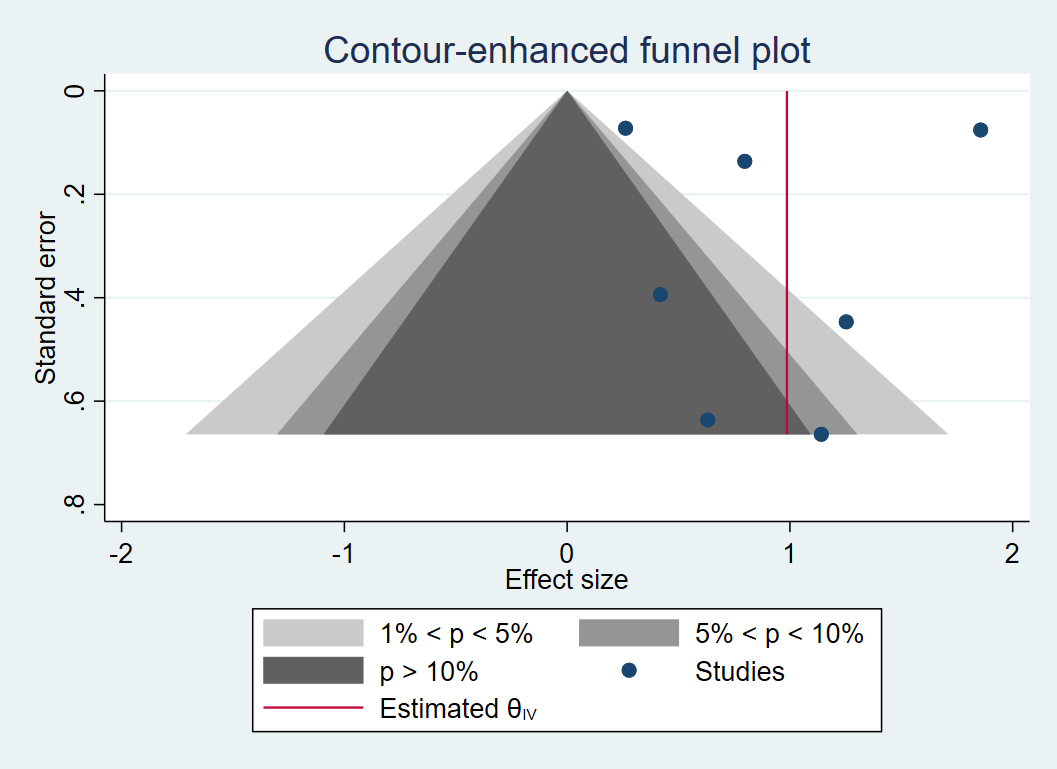
**

**Figure S2 IPV perpetrator; leave-one-out analysis**

**
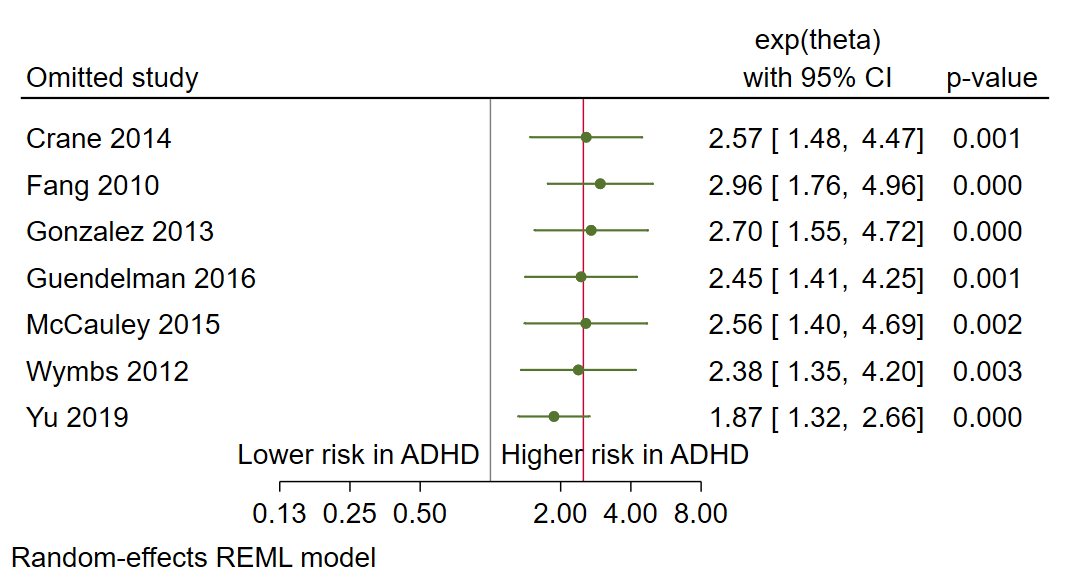
**

**Figure S3 IPV physical perpetrator; forest plot**

**
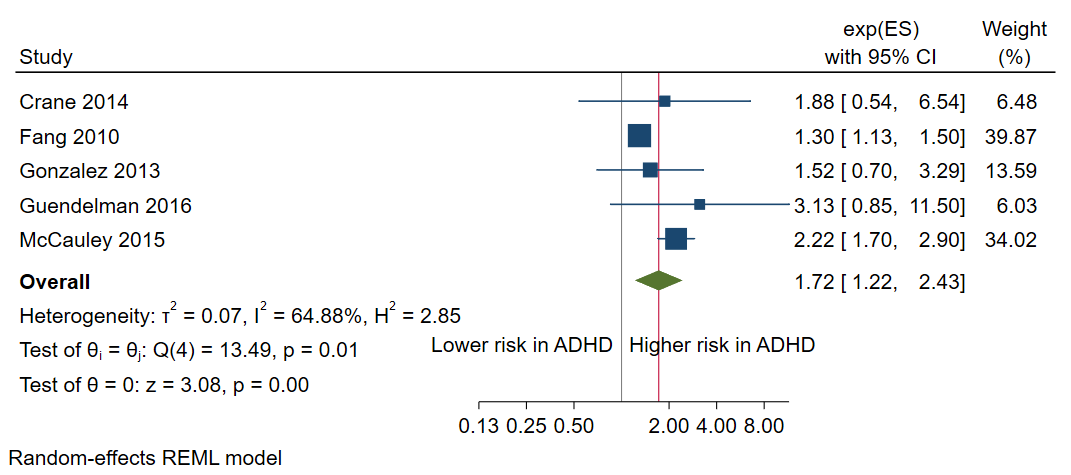
**

**Figure S4 IPV physical perpetrator; funnel plot**

**
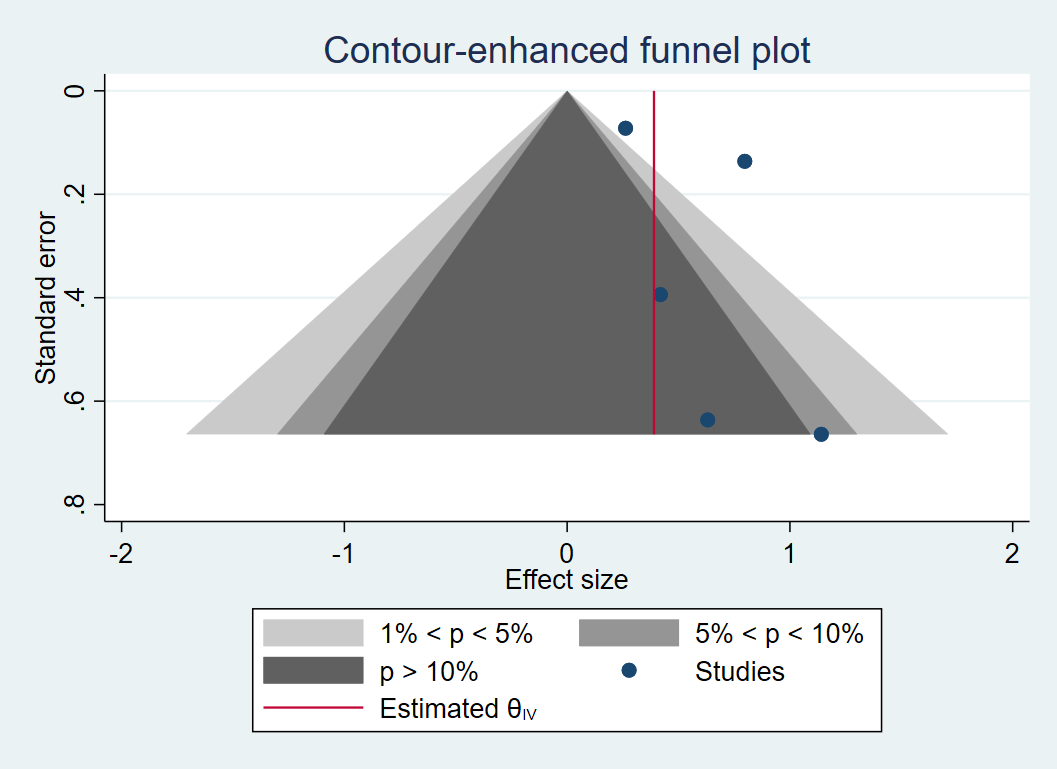
**

**Figure S5 IPV perpetrator; cross sectional; forest plot**

**
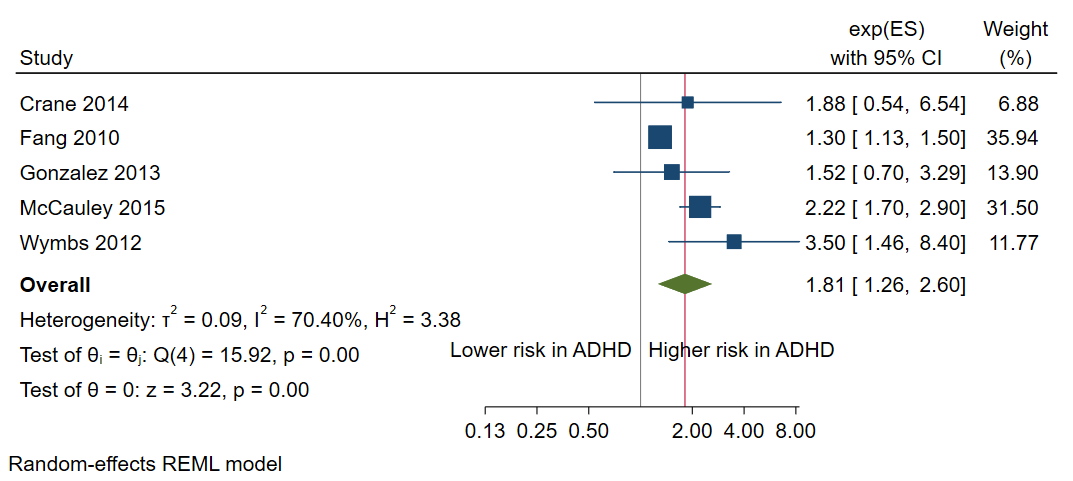
**

**Figure S6 IPV perpetrator; cross sectional; funnel plot**

**
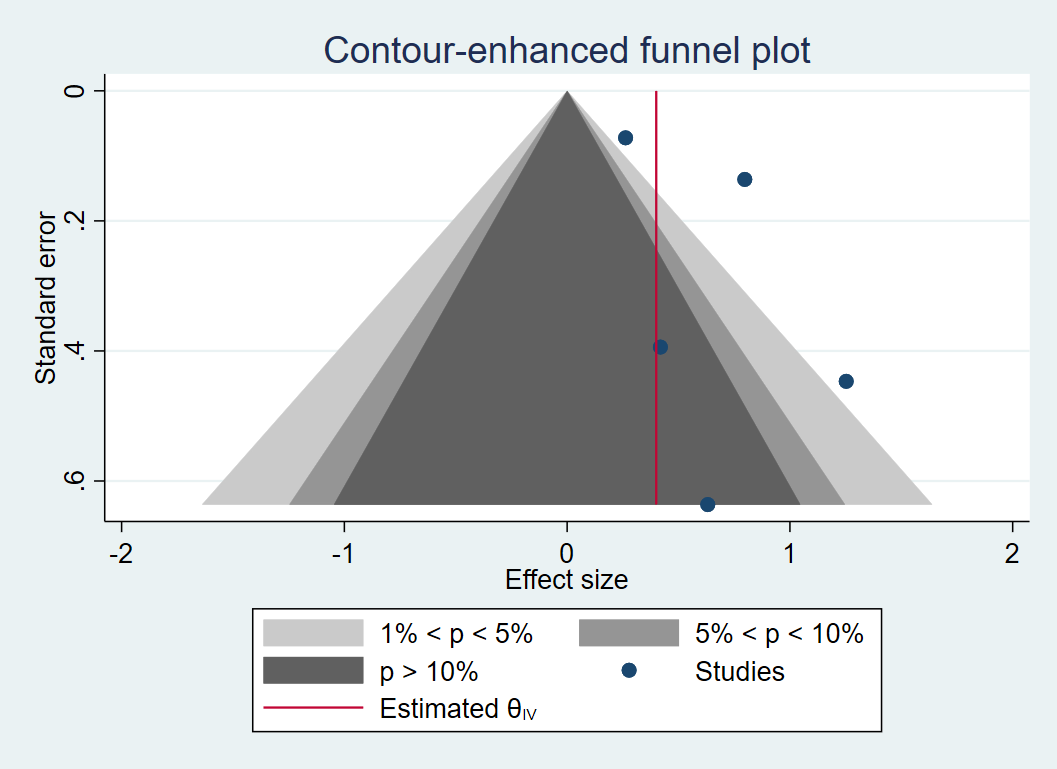
**

**Figure S7 IPV perpetrator; population-based; forest plot**

**
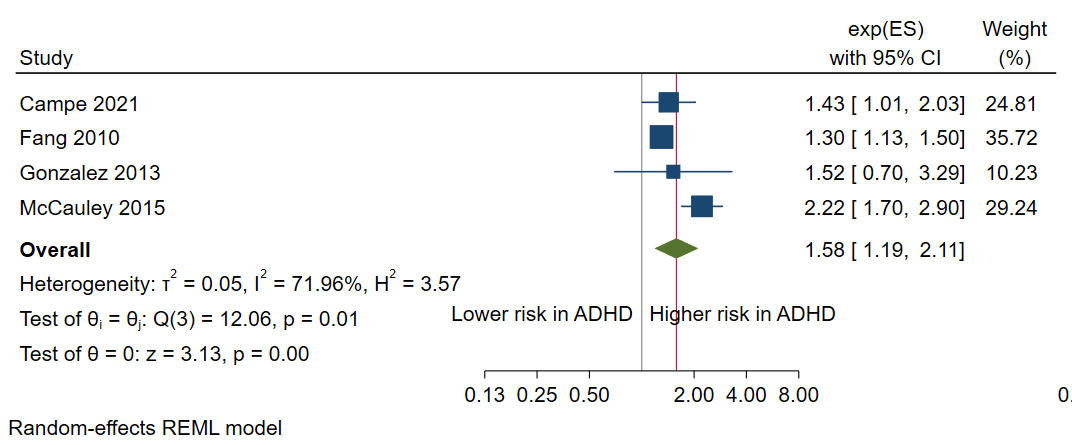
**

**Figure S8 IPV perpetrator; population-based; funnel plot**

**
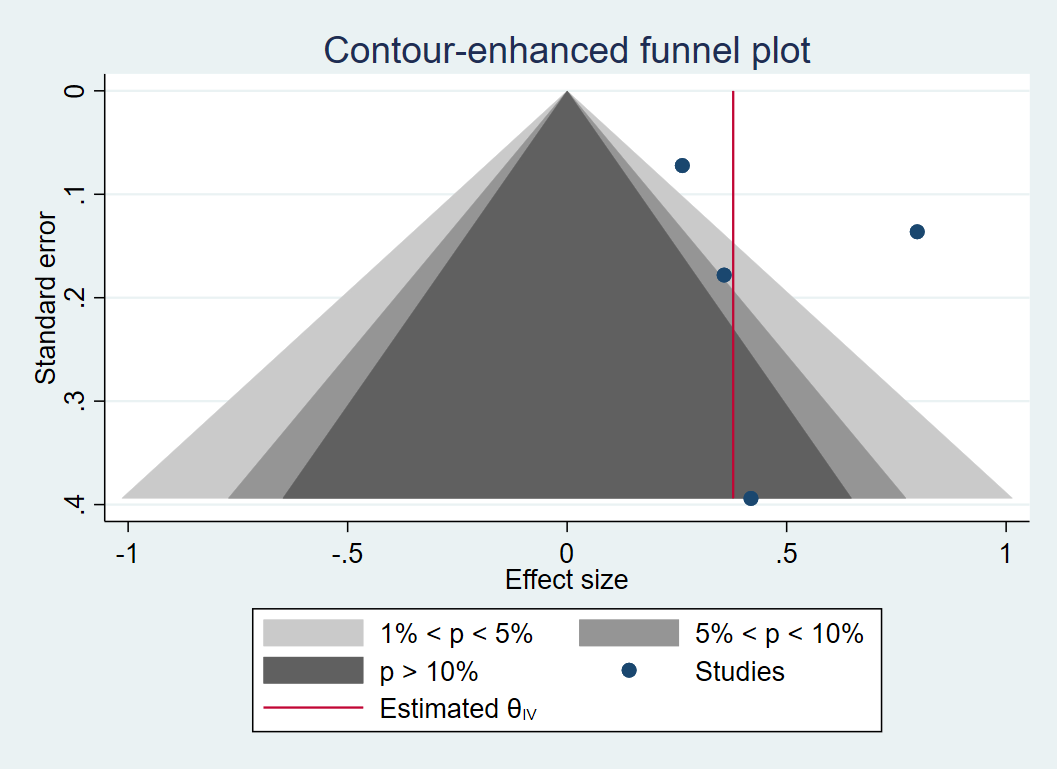
**

**Figure S9 IPV perpetrator; clinical or register-based diagnosis; forest plot**

**
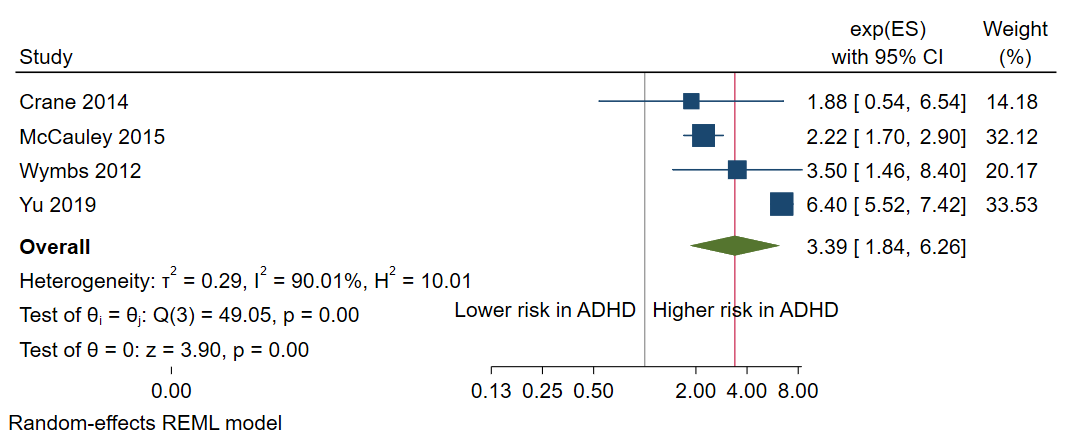
**

**Figure S10 IPV perpetrator; clinical or register-based diagnosis; funnel plot**

**
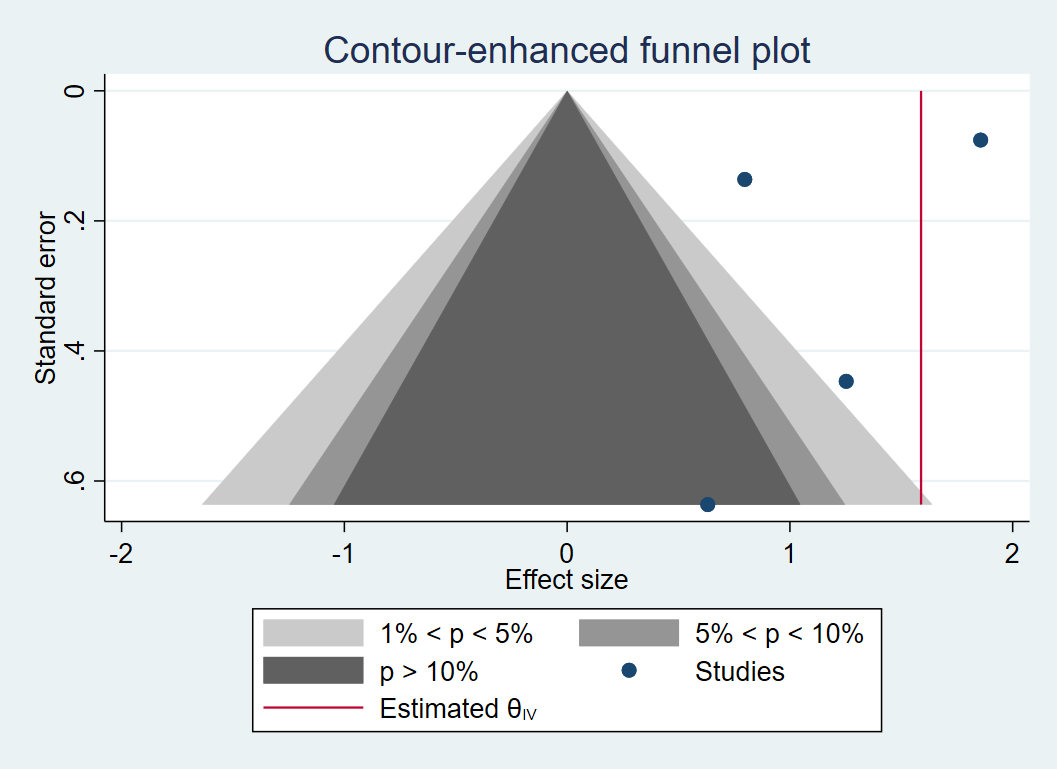
**

**Figure S11 IPV perpetrator; adjusted; forest plot**

**
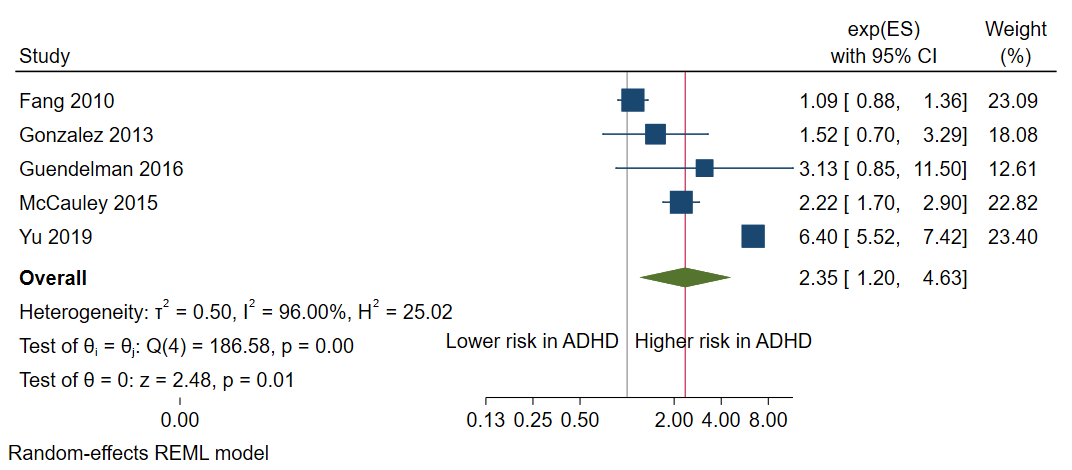
**

**Figure S12 IPV perpetrator; adjusted; funnel plot**

**
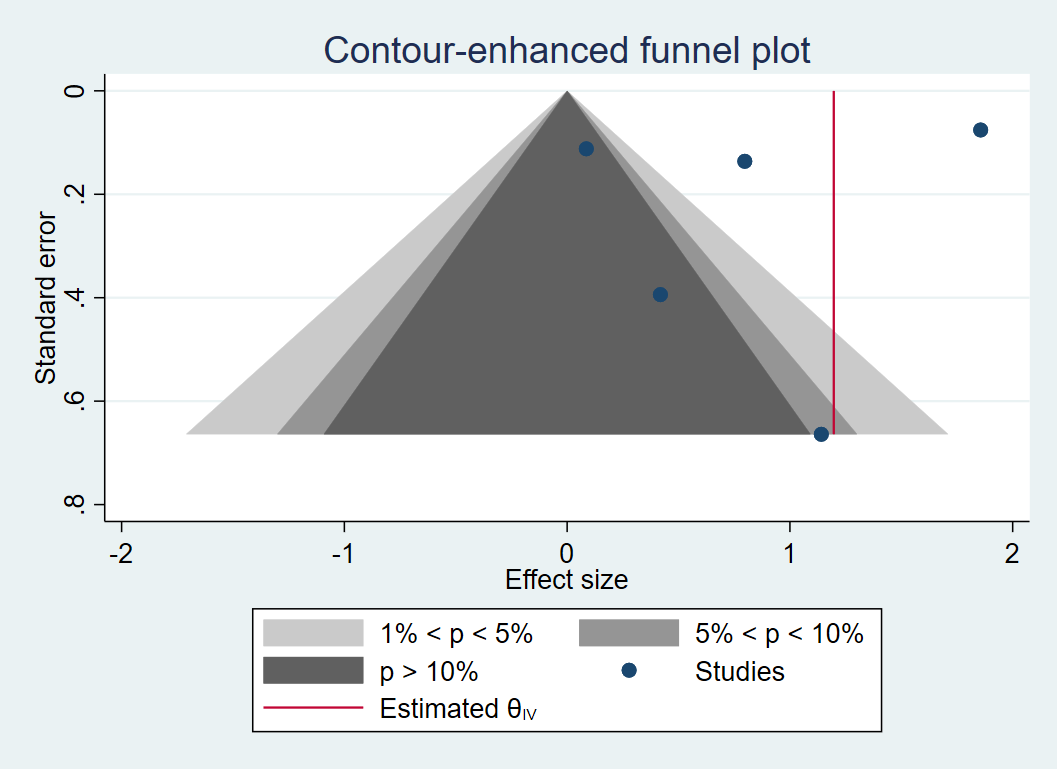
**

**Figure S13 IPV perpetrator; without studies deriving samples from the legal system; forest plot**

**
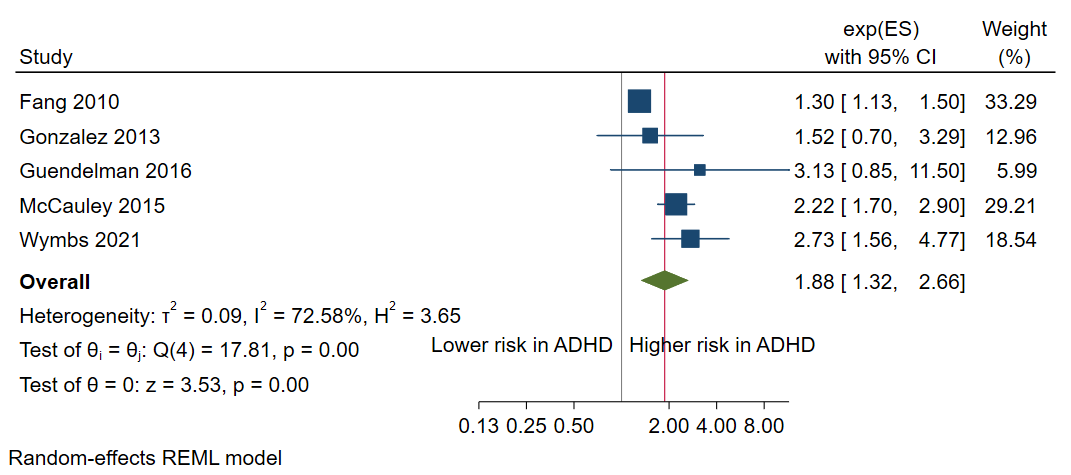
**

**Figure S14 IPV perpetrator; without studies deriving samples from the legal system; funnel plot**


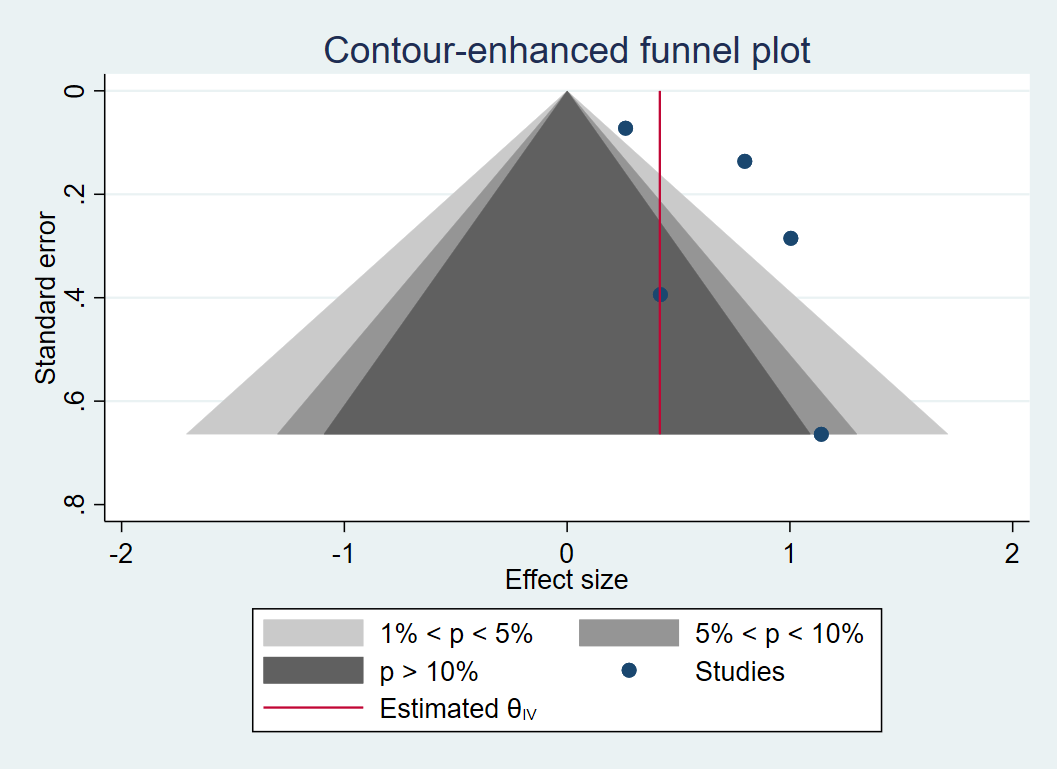


**Figure S15 SV perpetrator; funnel plot**

**
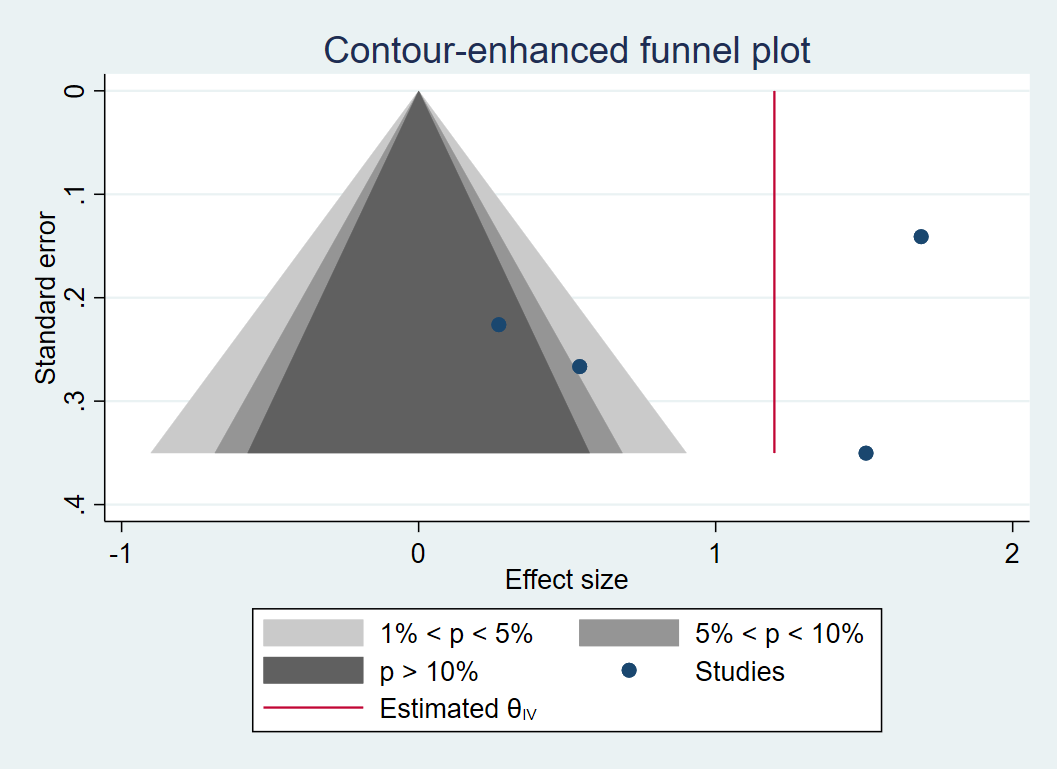
**

**Figure S16 SV perpetrator; leave-one-out analysis**

**
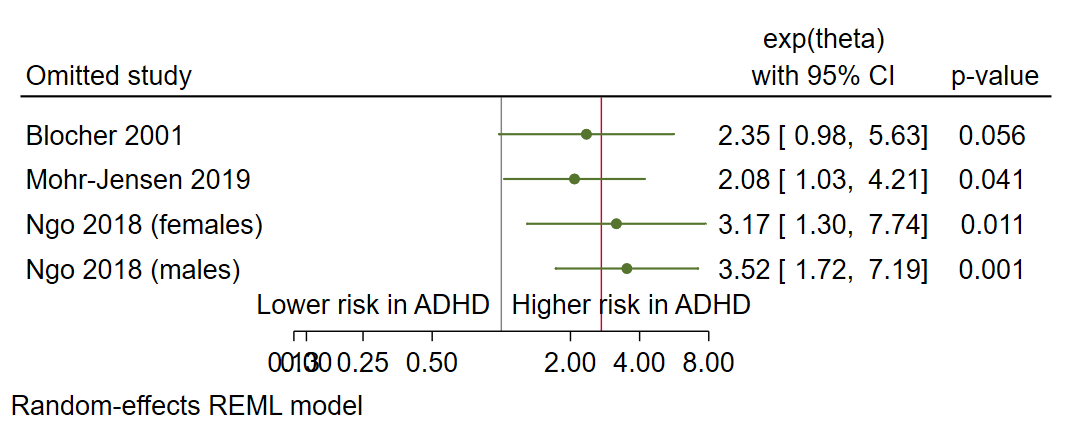
**

**Figure S19 IPV victim funnel; funnel plot**

**
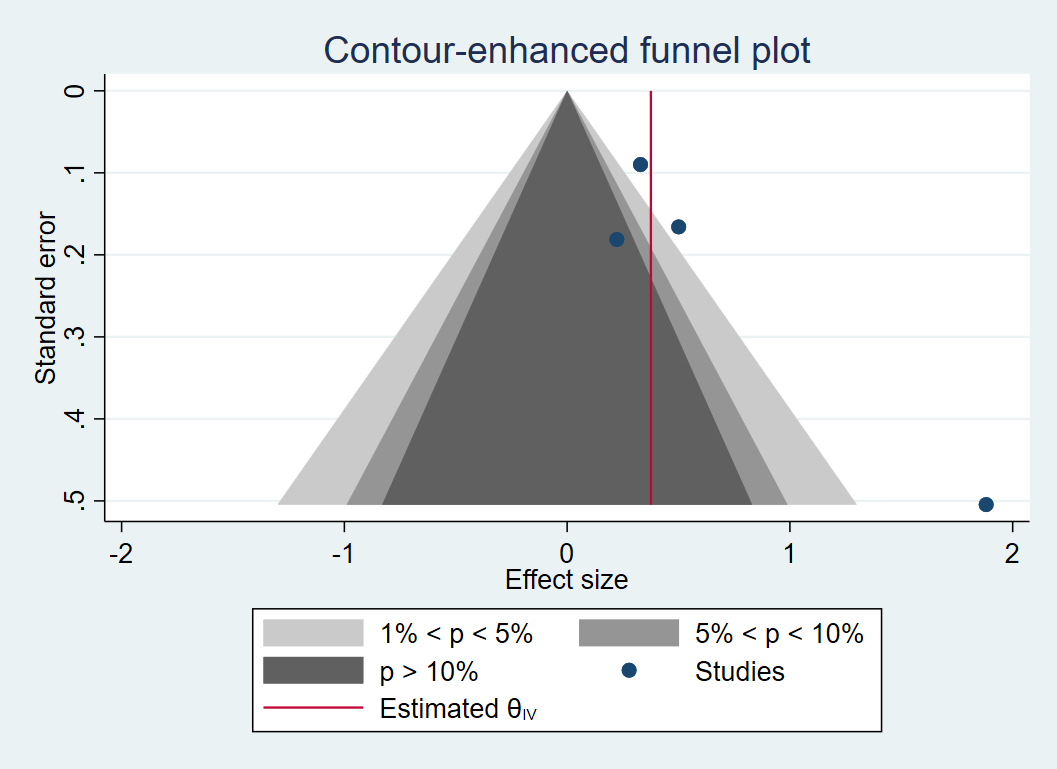
**

**Figure S20 IPV victim; leave-one-out analysis**

**
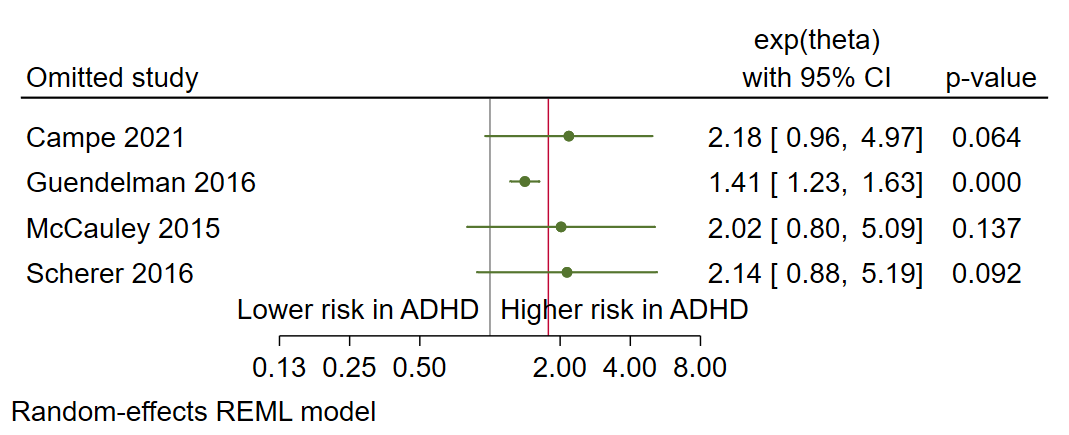
**

**Figure S21 IPV physical victim; forest plot**

**
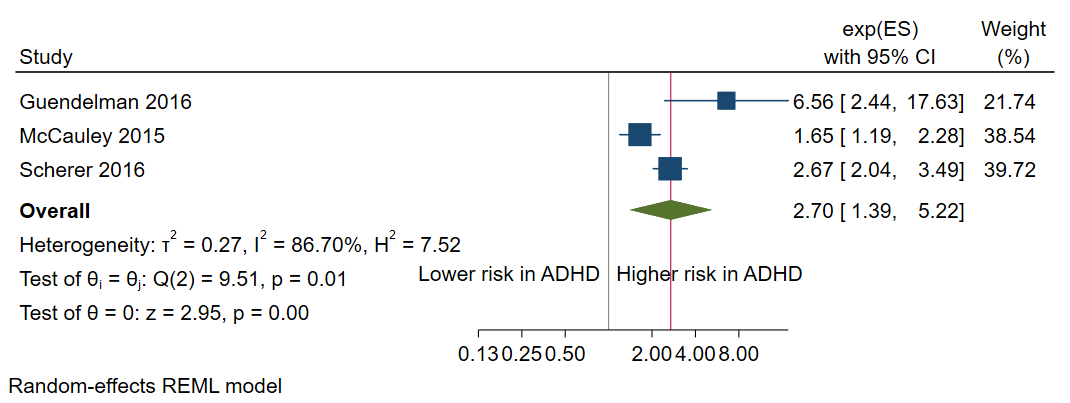
**

**Figure S22 IPV physical victim; funnel plot**

**
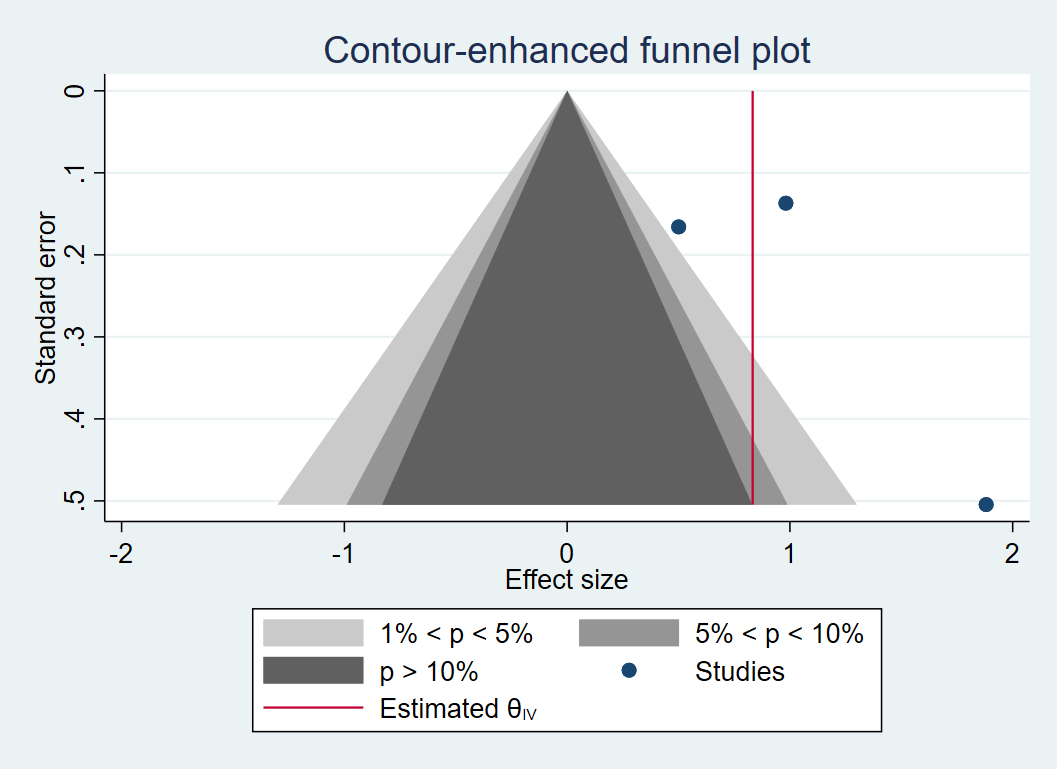
**

**Figure S23 IPV victim; adjusted; forest plot**

**
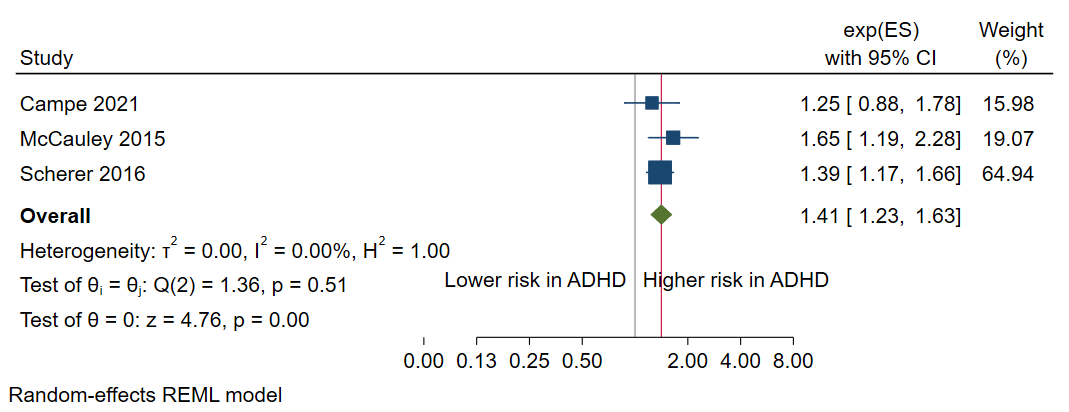
**

**Figure S24 IPV victim; adjusted; funnel plot**

**
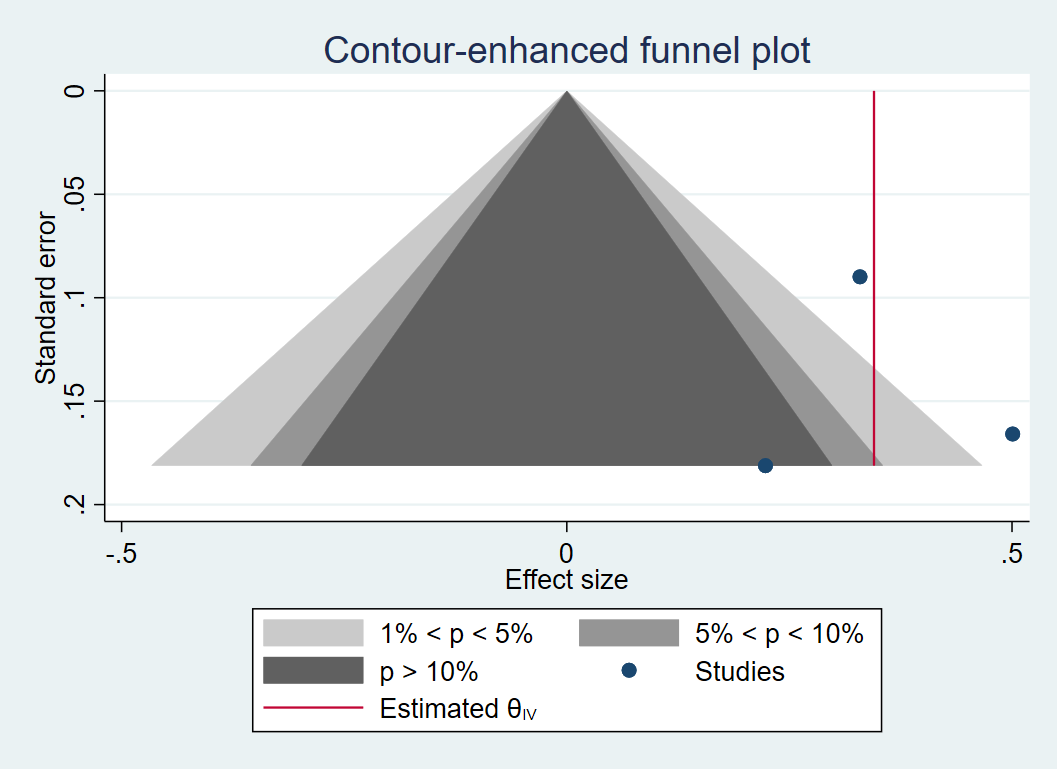
**

**Figure S25 SV victim funnel; funnel plot**

**
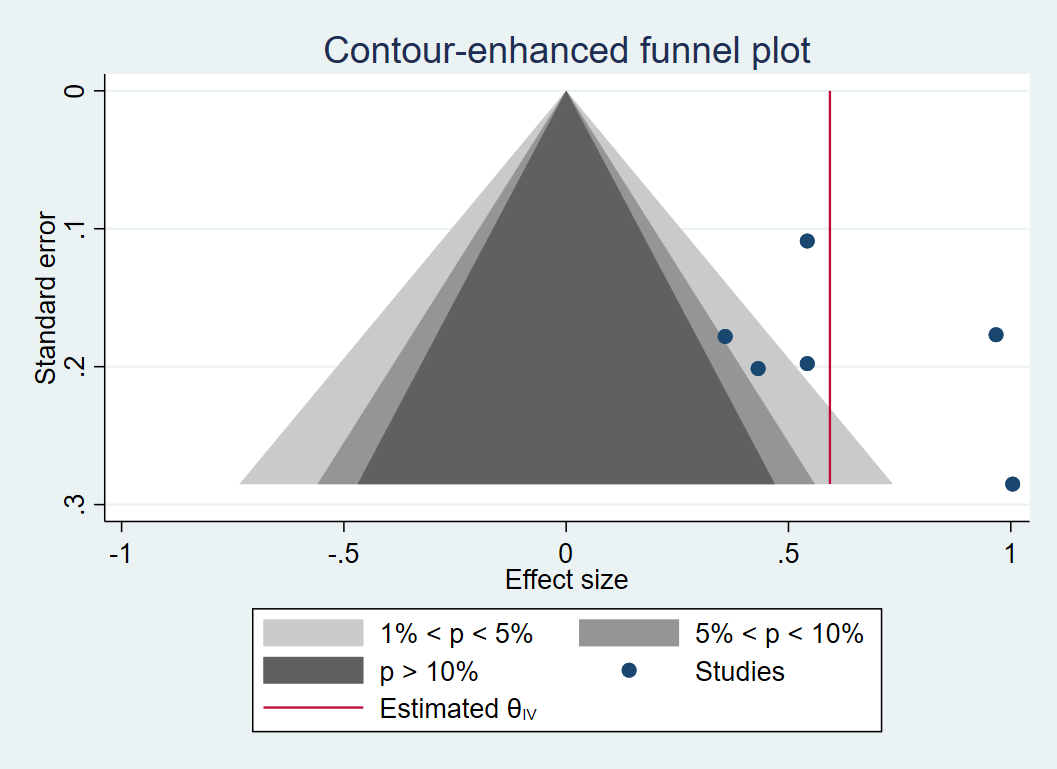
**

**Figure S26 SV victim; leave-one-out analysis**


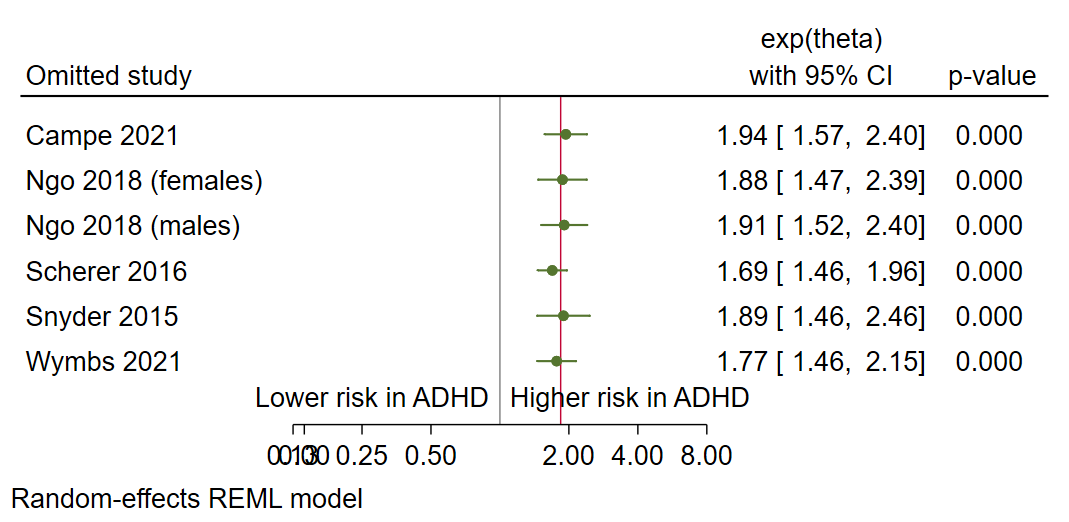


**Figure S27 SV victim; females; forest plot**

**
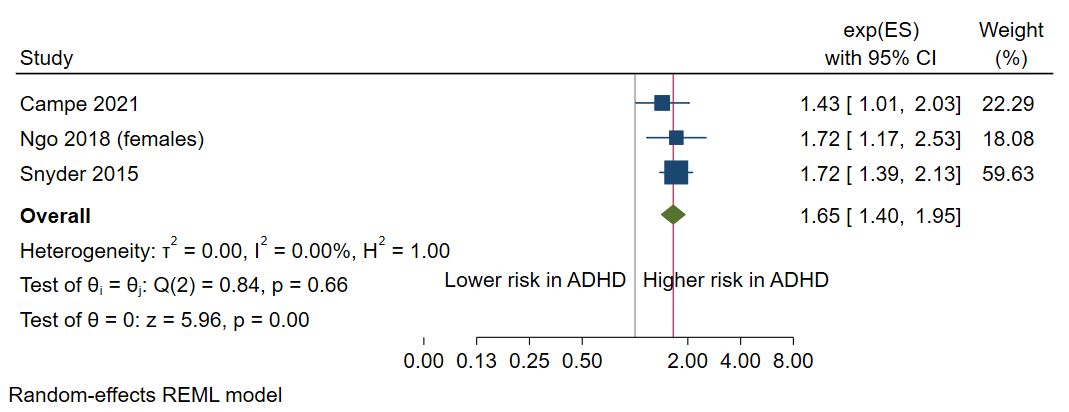
**

**Figure S28 SV victim; females; funnel plot**

**
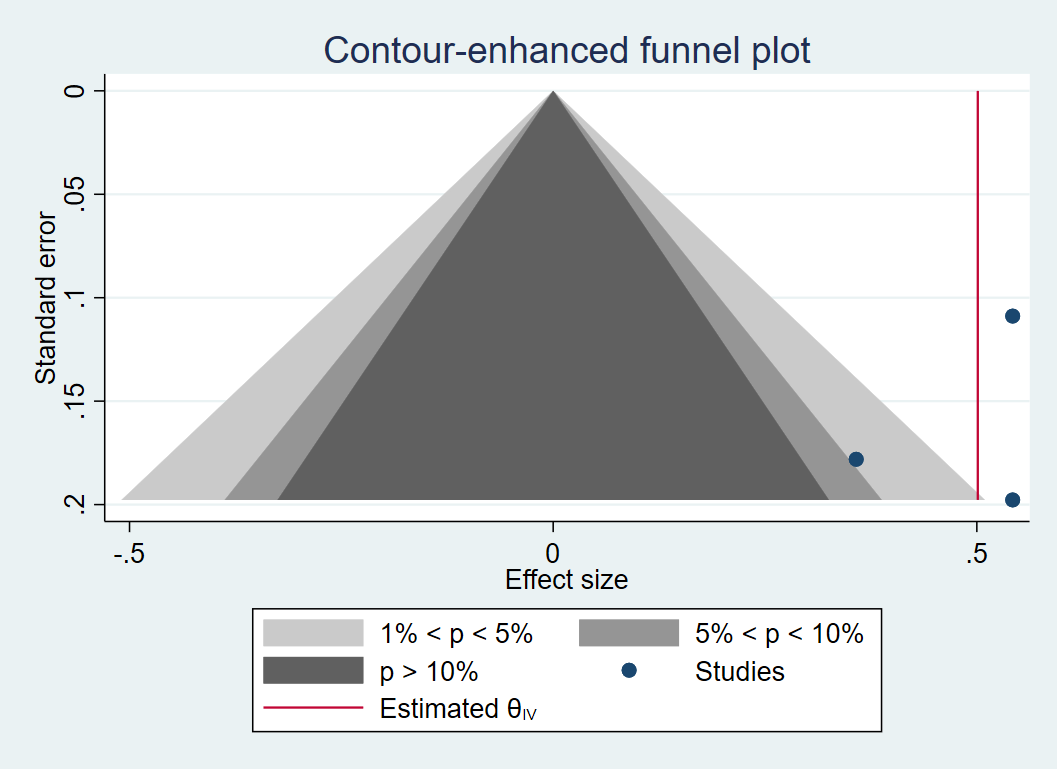
**

**Figure S29 SV victim; cross sectional; forest plot**

**
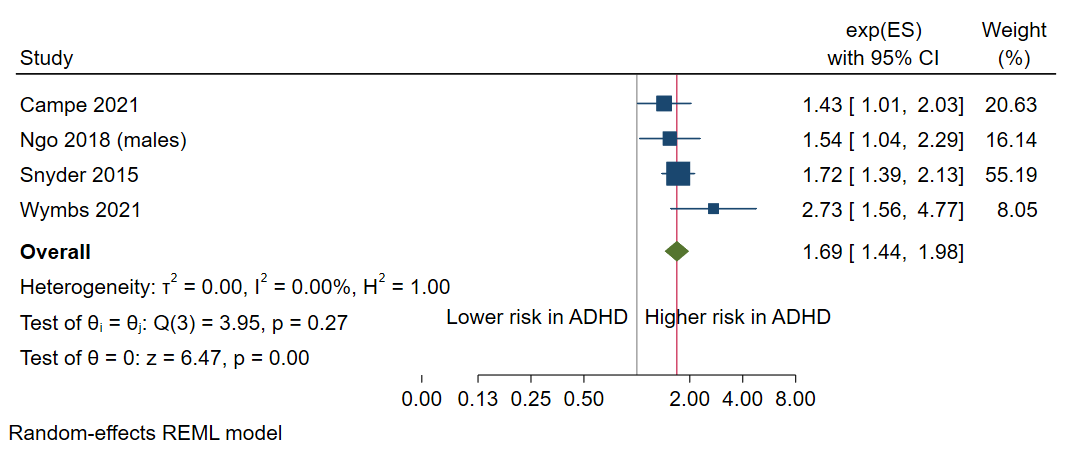
**

**Figure S30 SV victim; cross sectional; funnel plot**

**
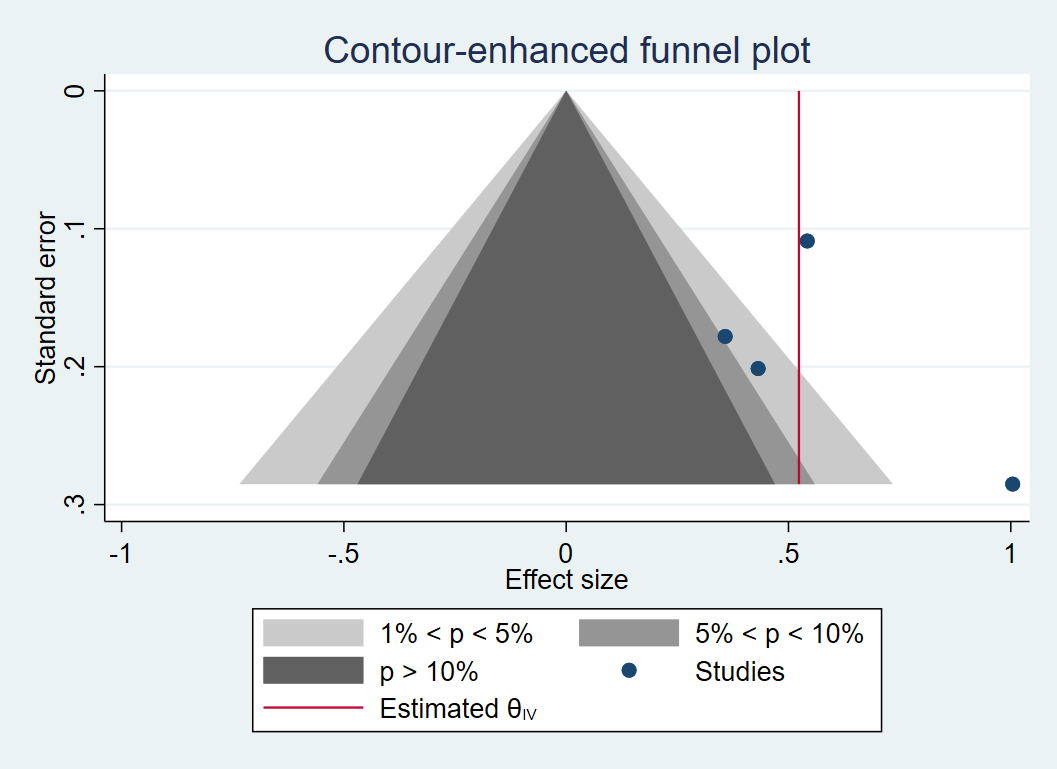
**

**Figure S31 SV victim; population-based; forest plot**

**
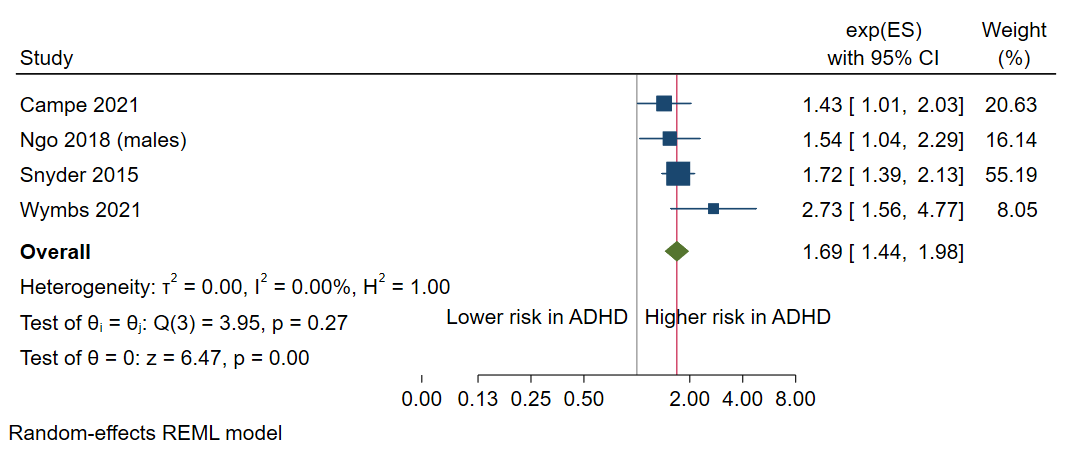
**

**Figure S32 SV victim; population-based; funnel plot**

**
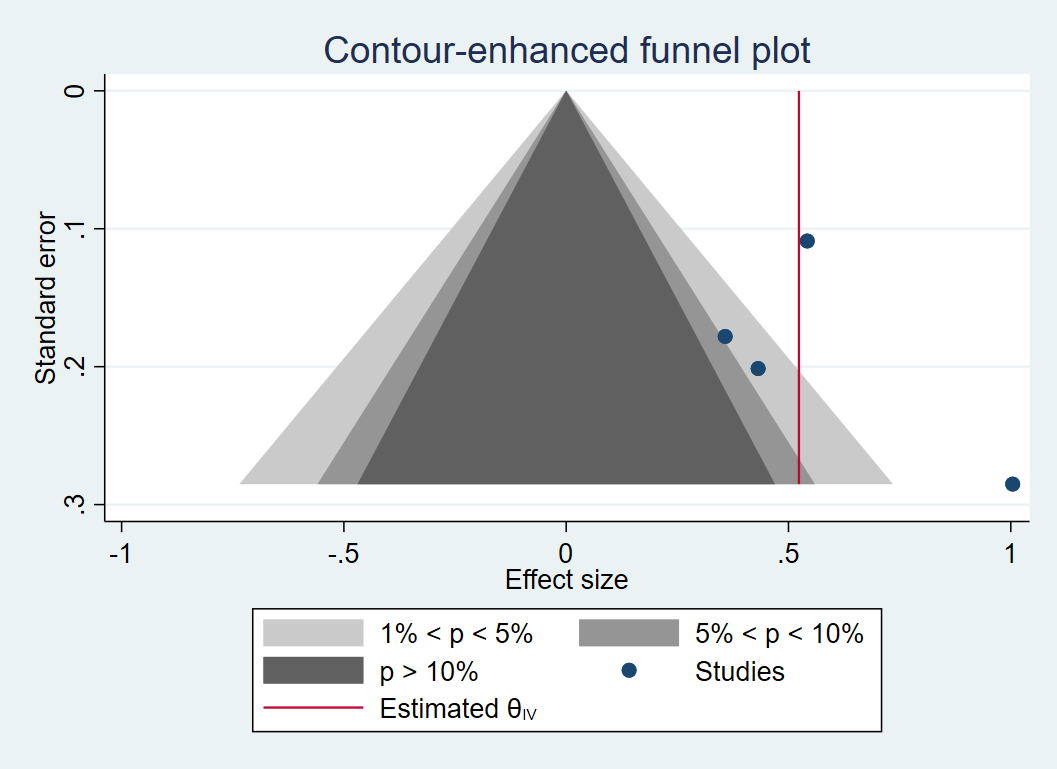
**

**Figure S33 SV victim; adjusted; forest plot**

**
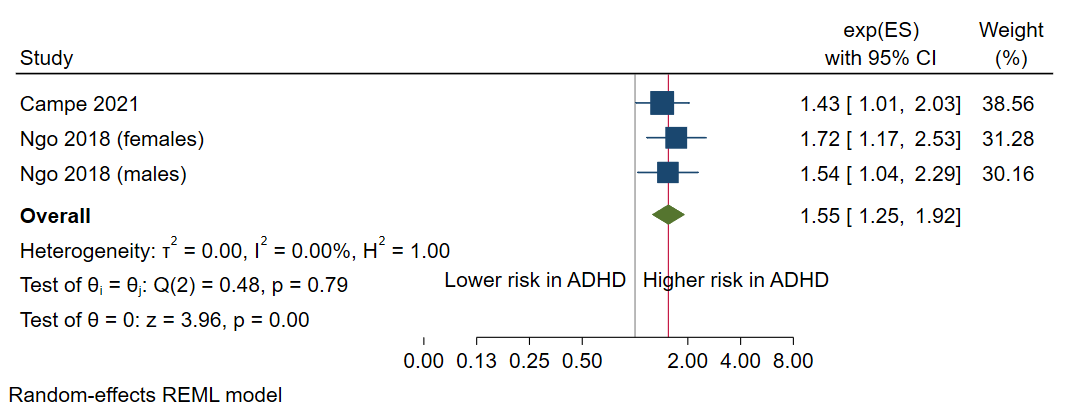
**

**Figure S34 SV victim; adjusted; funnel plot**


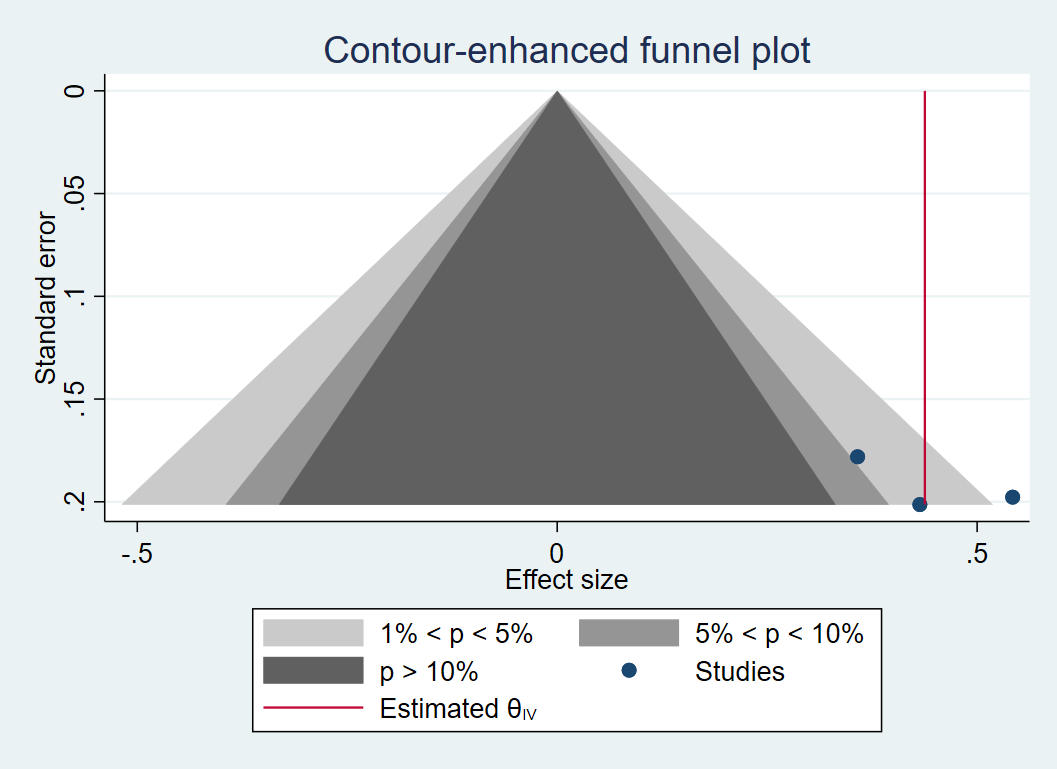

Supplement: Arrondo et al. supplementary material [file S0033291723001976sup001.docx]
